# Isolation and Characterization of Root-Associated Bacterial Endophytes and Their Biocontrol Potential against Major Fungal Phytopathogens of Rice (*Oryza sativa* L.)

**DOI:** 10.3390/pathogens9030172

**Published:** 2020-02-28

**Authors:** Maqsood Ahmed Khaskheli, Lijuan Wu, Guoqing Chen, Long Chen, Sajid Hussain, Dawei Song, Sihui Liu, Guozhong Feng

**Affiliations:** State Key Laboratory of Rice Biology, China National Rice Research Institute, Hangzhou 311400, China; khaskheli.maqsood89@gmail.com (M.A.K.); wulijuan@caas.cn (L.W.); chenguoqing@caas.cn (G.C.); chenlong@caas.cn (L.C.); hussainsajid@caas.cn (S.H.); 13051916600@163.com (D.S.); sihuil@126.com (S.L.)

**Keywords:** rice, root-associated endophytes, biological control, phytopathogens

## Abstract

Rice (*Oryza sativa* L.) is a major cereal food crop worldwide, and its growth and yield are affected by several fungal phytopathogens, including *Magnaporthe oryzae*, *Fusarium graminearum, F. moniliforme,* and *Rhizoctonia solani*. In the present study, we have isolated and characterized root-associated bacterial endophytes that have antifungal activities against rice fungal phytopathogens. A total of 122 root-associated bacterial endophytes, belonging to six genera (*Bacillus*, *Fictibacillus*, *Lysinibacillus*, *Paenibacillus*, *Cupriavidus*, and *Microbacterium*) and 22 species were isolated from three rice cultivars. Furthermore, the 16S rRNA sequence-based phylogeny results revealed that *Bacillus* was the most dominant bacterial genera, and that there were 15 different species among the isolates. Moreover, 71 root-associated endophytes showed antagonistic effects against four major fungal phytopathogens, including *M. oryzae*, *F. graminearum,* *F. moniliforme*, and *R. solani*. Additionally, the biochemical, physiological, and PCR amplification results of the antibiotic-related genes further supported the endophytes as potential biocontrolling agents against the rice fungal pathogens. Consequently, the findings in this study suggested that the isolated bacterial endophytes might have beneficial roles in rice defense responses, including several bioactive compound syntheses. The outcomes of this study advocate the use of natural endophytes as an alternative strategy towards the rice resistance response.

## 1. Introduction

Rice (*Oryza sativa* L.) is considered to be the most widely used staple food among the cereal crops, as it feeds half of the world’s population [1,2]. However, the average rice yield is less than its potential due to many biotic and abiotic factors [3]. Among the biotic factors, rice blast, rice seedling blight, rice bakanae, and rice sheath blight caused by *Magnaporthe oryzae*, *Fusarium graminearum, Fusarium moniliforme*, and *Rhizoctonia solani*, respectively, are the common fungal phytopathogenic diseases causing severe crop damage and losses in yield [4,5,6,7,8]. The annual rice grain yield losses due to major fungal diseases were reported to be rice blast (30%), seedling blight, rice bakanae (50%–60%), and sheath blight (40%–50%) in Asian countries [7,9,10]. At present, the application of fungicides is considered to be the most practiced way of mitigating these fungal diseases of rice. However, the focus has been given to the effective biocontrol of these phytopathogens, as the use of excessive fungicides and chemicals can cause pathogen resurgence and other environmental hazards [11,12]. Several biocontrol approaches have been made to control the respective rice diseases to date [13], of which, the use of root-associated bacterial endophytes has proven to be highly effective. The root-associated bacterial endophytes are responsible for the mechanisms of antagonistic activities against plant fungal phytopathogens by the production of antibiotics, hydrogen cyanide, lytic enzyme activity, catalase, and siderophore [14]. Furthermore, the central role of root-associated endophytes is to prevent phytopathogenic influence and to create competitiveness by producing colonization with the availability of minerals and nutrients [15]. This biocontrol approach is environmentally friendly and effective against biotic and disease stresses. For example, the *Pseudomonas chlororaphis* EA105 endophytic microbe discovered from rice roots mitigates the disease-causing effect to *M. oryzae* in vitro and in vivo. The role of *P. chlororaphis* EA105 has been extensively studied for its possible use as a biological control and the ecological benefits in rice [16,17,18].

The bacterial endophytes (*Bacillus pumilus sp., Bacillus subtilis sp.,* and *Pseudomonas fluorescence sp.*) have been used to select the microorganisms, through in vitro screening on dual-culture agar plates, where the microbes showed antagonistic abilities against many seed- and soil-borne rice fungal phytopathogens [19,20]. These bacterial species have the characteristics to produce antifungal metabolites and to protect crop plants from fungal infection [21]. Additionally, in modern agriculture, the application of root-associated bacterial endophytes is a prime option, with better antagonistic effects as a biocontrol agent [22]. However, the proper method for the collection of root-associated bacterial endophytes from the various control conditions, as well as its usage as a biocontrol agent against fungal phytopathogens, is largely unknown [23]. Moreover, the molecular screening of antibiotic encoding genes for particular endophytic bacteria, which are responsible for the number of antibiotics and antifungal metabolites production, including cyclic lipopeptides, bio-surfactin, 2, 4-di-acetyl phloroglucinols, pyoluteorin, phenazines, bacillomycins, fengycin, lipopeptides, pyrrolnitrin, celluloses, hydrogen cyanide, polyketide synthase, and non-ribosomal peptide synthases has been considered to be a valuable option for beneficial endophyte selection. These are the most promising bioactive compounds that are associated with biocontrol mechanisms against a wide range of bacterial and fungal phytopathogens [24,25]. The isolation and identification of these endophytic bacteria are essential for agricultural manipulations [26]. 

Keeping in view the above mentioned important points, the objectives of the present study are to identify and isolate root-associated bacterial endophytes from rice root tissues, by using morphological and molecular approaches to assess the biotic stress problems, and to check their disease suppressing abilities with antagonistic activities for a broad spectrum host range. 

## 2. Results

### 2.1. Isolation and Identification of Root-Associated Bacterial Endophytes 

We successfully isolated 122 culture-able root-associated bacterial endophyte strains based on their visual morphological characterizations from the surface-sterilized roots of available rice cultivars in the field, such as Xiushui-48, Y-003, and CO-39. The isolation frequencies of these bacterial endophytes were further demonstrated on the Luria Brentani agar medium (LB). Among these bacterial endophytes, 45 were conformed from the Xiushui-48 cultivar (C-1), 40 endophytes from the Y-003 cultivar (C-2), and 37 endophytes were detected from the CO-39 cultivar (C-3) (Table 1).

### 2.2. Molecular Classification and 16S rRNA Gene Sequences Based Phylogeny Association of Root-Associated Bacterial Endophytes 

**Figure 1:**
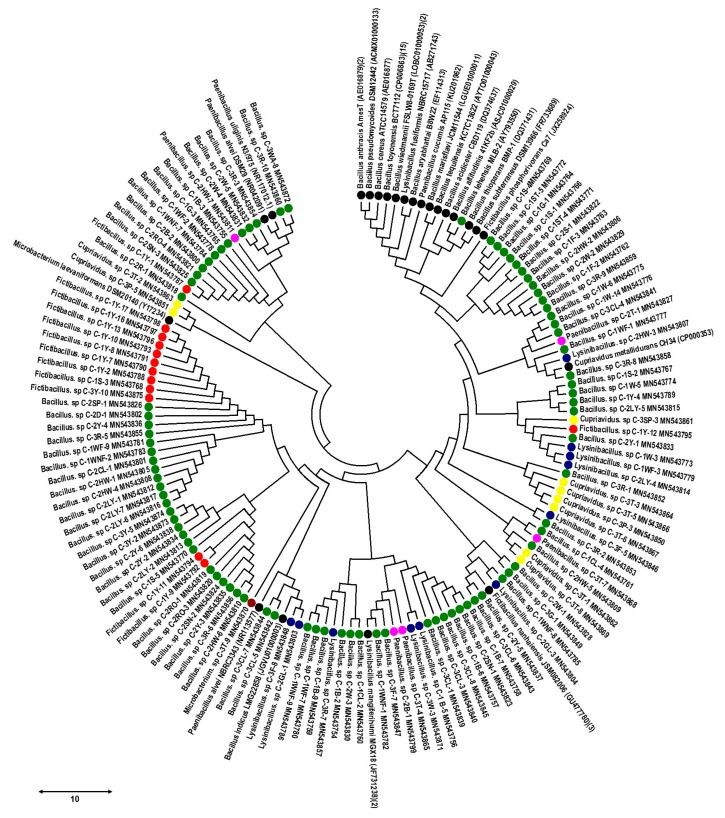
The 16S rRNA gene sequences-based phylogeny of all the 122 root-associated bacterial endophytes isolated from different rice cultivars. The 16S rRNA gene sequences were clustered to the MUSCLE alignment through the neighbor-joining method and the 16S rRNA gene sequences-based phylogeny was concatenated by inputting the 16S rRNA gene sequences into the MEGA 7 software. The above green, red, blue, yellow, pink, dark red and black, colored circle symbols represent different bacterial species such as; *Bacillus ssp., Fictibacillus ssp., Lysinibacillus ssp., Cupriavidus ssp., Paenibacillus ssp., Microbacterium ssp.,* and reference type strains. The scale bar value indicates to the bootstrap analysis from (1000) replicates for the genetic distance.

The initially isolated 122 root-associated bacterial endophytes were confirmed by molecular characterization through the 16S rRNA gene sequences for the identification of bacterial species. Furthermore, these bacterial endophytes were identified at a high threshold level of about ≥99%–100% compared with most associated bacterial type strains. The screened bacterial endophytes sequence data were submitted to the NCBI GenBank, under the following accession numbers: MN543754 to MN543875. Based on the 16S rRNA gene, the results showed that the 122 cultured root-associated bacterial endophytes were highly associated with six genera (*Bacillus*, *Fictibacillus*, *Lysinibacillus*, *Paenibacillus*, *Cupriavidus*, and *Microbacterium*). These genera were sub-divided into the 22 different bacterial species separately, and the *Bacillus* genera were the most dominant ones within the isolated root-associated bacterial endophyte. Moreover, among the isolates, the most abundant species in the root-associated bacterial endophytes were *Bacillus altitudinis ssp.* (28 isolates), *Bacillus aryabhattai ssp.* (16 isolates), *Bacillus cereus ssp.* (12 isolates), *Bacillus fusiformis ssp.* (9 isolates), *Fictibacillus phosphorivorans ssp.* (9 isolates), and *Cupriavidus metallidurans ssp.* (9 isolates) respectively. Among the remaining isolates, the bacterial endophyte species were found with different numbers of isolates, such as *Bacillus marisflavi ssp.* (8 isolates), *Bacillus wiedmannii ssp.* (8 isolates), *Fictibacillus nanhaiensis spp.* (4 isolates), *Bacillus subterraneous spp.* (3 isolates), *Bacillus thioparans ssp.* (2 isolates), *Lysinibacillus mangiferihumi ssp.* (2 isolates), *Paenibacillus cucumis ssp.* (2 isolates), and *Paenibacillus alvei ssp.* (2 isolates) respectively. Whilst other bacterial endophytes i.e., *Bacillus pseudomycoides ssp., Bacillus lehensis ssp., Bacillus acidiceler ssp., Bacillus tequilensis ssp., Bacillus indicus ssp., Bacillus toyonensis ssp., Paenibacillus uliginis ssp.,* and *Microbacterium laevaniformans ssp.* were found with one strain. Further, these 122 root-associated bacterial endophyte strains, identified by the 16S rRNA gene sequences relationship with the best match type strains (T) from the NCBI database, and the 16S rRNA gene sequences-based phylogeny are presented in Table 1 and Figure 1**.**

### 2.3. Biochemical and Physiological Characterization of Root-Associated Bacterial Endophytes

The in-vitro screening results were verified and summarized for the 122 root-associated bacterial endophytes, with respect to their biochemical and physiological traits, with positive and negative symbol indications given in Table S2. Among these 122 root-associated bacterial endophytes, 112 bacterial endophytes showed gram-positive reactions (GPR) and 10 isolates showed gram-negative reactions (GNR). The classification on the basis of their role in different activities, i.e., catalase activity (CA), endospore activity (EA), and oxidase activity (OA), where 104, 96, and 102 isolates showed positive reactions, and 18, 26, and 20 bacterial isolates had negative reactions, respectively. Similarly, 71, 72, and 92 isolates showed positive reactions, and 51, 50, and 30 showed negative reactions, regarding motility performance (MP), citrate activity (CA), and gelatinase production (GP), respectively. Moreover, 110 isolates showed a positive response, and 12 isolates showed a negative reaction, to urease activity (UA), 64 isolates showed a positive reaction and 58 isolates showed a negative reaction in nitrate reduction activity (NRA). There were 98 positive and 24 negative reactions related to the Voges–Proskauer performance (VPP). Furthermore, 113 isolates showed a positive reaction and 9 isolates showed a negative reaction for the methyl red activity (MRA). For starch hydrolyze activity (SHA), 94 bacterial endophytes expressed a positive reaction and 28 isolates had a negative reaction. We found 100 isolates with a positive reaction and 22 isolates with a negative reaction for the glucose activity (GA). With concern to the mannitol activity (MA), lactose activity (LA), and maltose activity (MA), 113, 110, and 84 bacterial endophytes were found as positive and 9, 12, and 38 isolates had a negative response, respectively.

### 2.4. In-vitro Antagonistic Activities of Root-Associated Bacterial Endophytes

**Figure 2:**
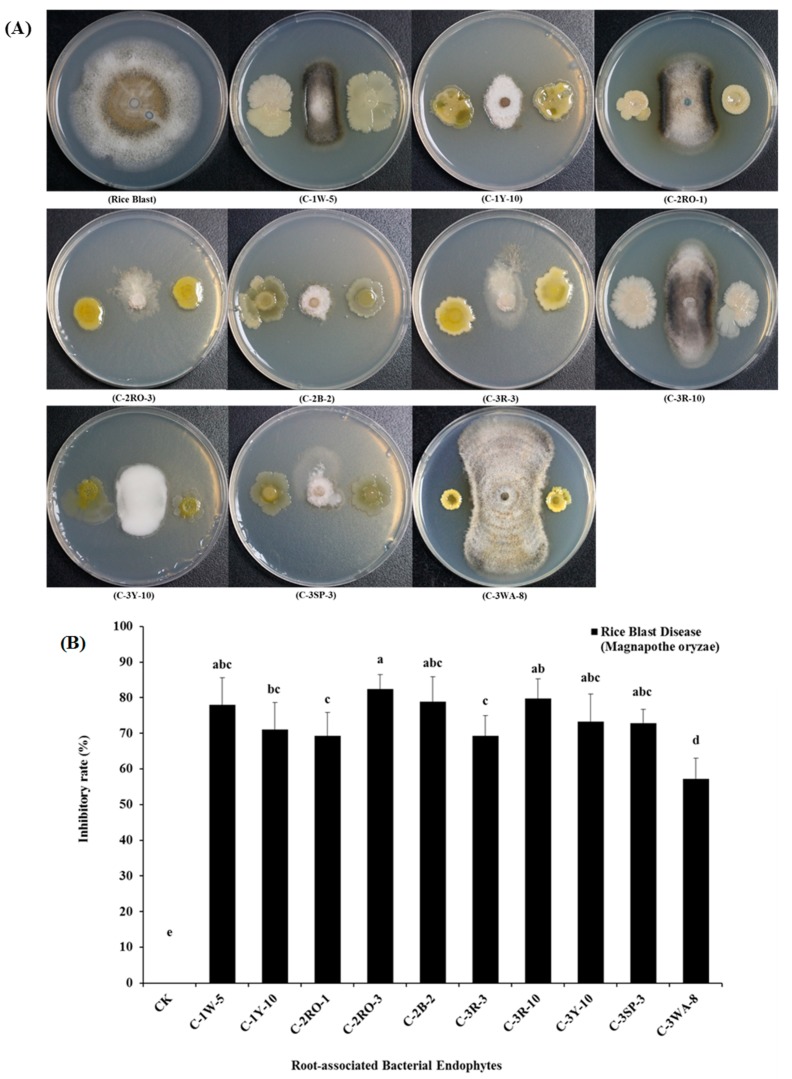
Biocontrol potentials of different root-associated bacterial endophytes to rice blast (*Magnaporthe oryzae*). (**A**) The above plates represent in-vitro antifungal inhibitory activities by dual-culture antagonistic tests of different root-associated bacterial endophytes against rice blast (*Magnaporthe oryzae*) phytopathogen. (**B**) The above selected graphical bars indicate antifungal inhibition percentages of radial mycelial growth of (*Magnaporthe oryzae*) by the 10 root-associated bacterial endophytes, respectively. Values are denoted as mean ± SE (*n=3*). Values followed by different letters are significantly different (p≤0.05) according to the least significant difference (LSD) test.

The in-vitro dual-culture test has been extensively used for preliminary screening of biocontrol agents against several fungal phytopathogens. In this study, a total of 122 root-associated bacterial endophytes were isolated using the in-vitro antagonistic tests. However, from these bacterial endophytes, the following 10 strains i.e., C-1W-5, C-1Y-10, C-2RO-1, C-2RO-3, C-2B-2, C-3R-3, C-3R-10, C-3Y-10, C-3SP-3, and C-3WA-8 belonged to the *Bacillus altitudinis ssp., Fictibacillus phosphorivorans ssp.,* and *Cupriavidus metallidurans ssp.* These strains were exhibited very strong antifungal antagonistic potential with varying amounts of mycelial inhibitory rates ranging from 57.2% to 82.4%, under dual-culture assay subjected to the rice blast (*Magnaporthe oryzae*) pathogen (Figure 2A,B). 

**Figure 3:**
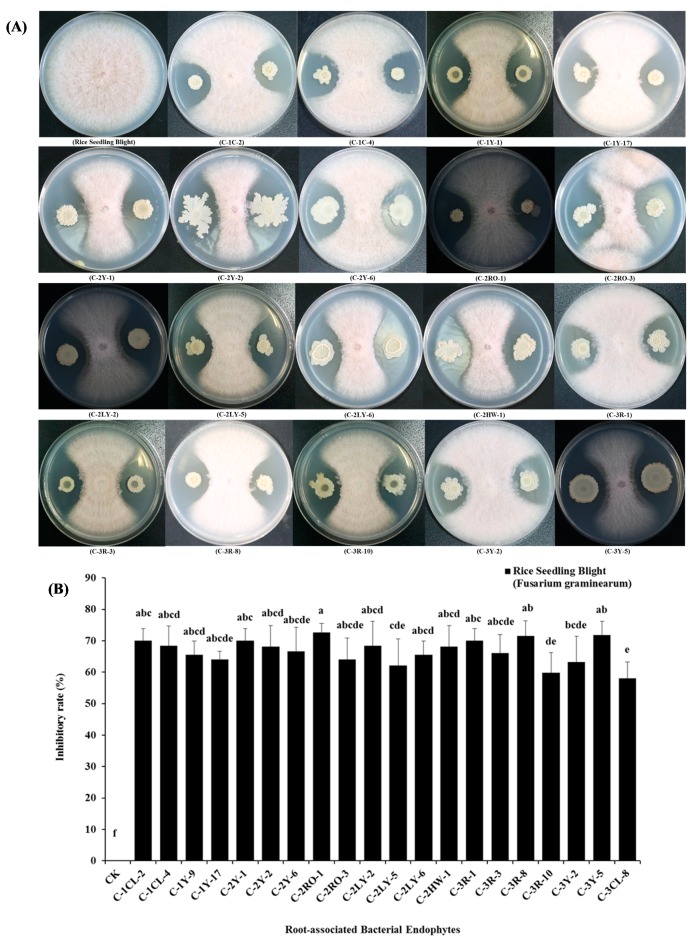
Biocontrol potential of different root-associated bacterial endophytes to rice seedling blight (*Fusarium graminearum*), (**A**) the above plates represent in-vitro antifungal inhibitory activities by dual-culture antagonistic tests of different root-associated bacterial endophytes against rice seedling blight (*Fusarium graminearum*) phytopathogen, (**B**) the above selected graphical bars indicate antifungal inhibition percentages of radial mycelial growth of (*Fusarium graminearum*), by the 20 root-associated bacterial endophytes respectively. Values are denoted as mean ± SE (*n = 3*). Values followed by different letters are significantly different (*p* ≤ 0.05) according to the LSD test.

Similarly, about 20 isolates strains (i.e., C-1CL-2, C-1CL-4, C-1Y-9, C-1Y17, C-2Y-1, C-2Y-2, C-2-Y-6, C-2RO-1, C-2RO-3, C-2LY-2, C-2LY-5, C-2LY-6, C-2HW-1, C-3R-1, C-3R-3, C-3R-8, C-3R-10, C-3Y-2, C-3Y-5, and C-3CL-8) among all bacterial endophytes were validated with high antagonistic activities against the mycelia growth (*Fusarium graminearum*), a fungal pathogen of rice seedling blight. Their inhibitory growth zones ranged from 58.0% to 72.6% of the respective pathogen under the dual-culture test results. Further, these 20 root-associated bacterial endophytes are closely associated with the *B. altitudinis ssp., B. wiedmannii ssp., B. aryabhattai ssp., B. marisflavi ssp., B. indicus ssp.,* and *F. phosphorivorans ssp.,* respectively (Figure 3A,B).

**Figure 4:**
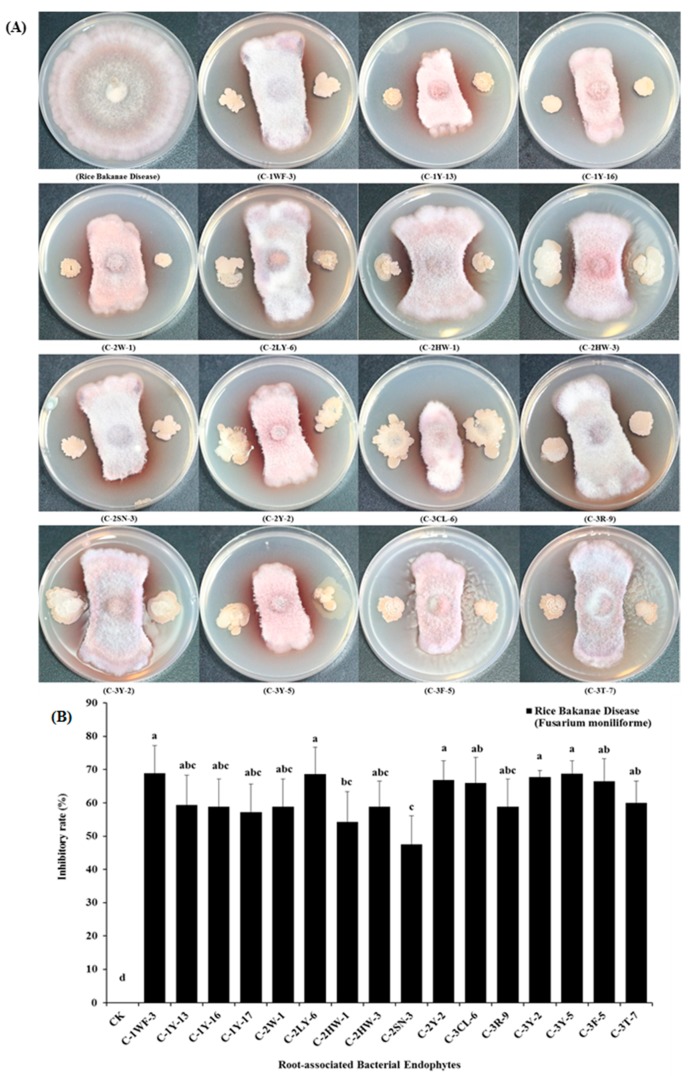
Biocontrol potential of different root-associated bacterial endophytes to rice bakanae (*Fusarium moniliforme*), (**A**) the above plates represent in-vitro antifungal inhibitory activities by dual-culture antagonistic tests of different root-associated bacterial endophytes against rice bakanae (*Fusarium moniliforme*), fungal phytopathogen of rice, (**B**) the above selected graphical bars indicate antifungal inhibition percentages of radial mycelial growth of (*Fusarium moniliforme*) by the 16 root-associated bacterial endophytes respectively. Values are denoted as mean ± SE (*n = 3*). Values followed by different letters are significantly different (*p* ≤ 0.05) according to the LSD test.

Moreover, 16 root-associated bacterial endophyte strains (i.e., C-1WF-3, C-1Y-13, C-1Y-16, C-1Y17, C-2W-1, C-2LY-6, C-2HW-3, C-2SN-3, C-2Y-2, C-3CL-6, C-3R-9, C-3Y-2, C-3Y-5, C-3F-5, and C-3T-7) among the total isolates were capable of displaying antagonistically biocontrol activities with the presence of significant inhibitory rates, from 47.5% to 68.9%, against the mycelia (*Fusarium moniliforme*) growth of rice bakanae. Additionally, the antifungal detected root-associated endophytes belong to various bacterial species, i.e., *L. fusiformis ssp., L. mangiferihumi ssp., F. phosphorivorans ssp., B. altitudinis ssp., B. marisflavi ssp., B. cereus ssp., B. aryabhattai ssp.,* and *P. cucumis ssp.,* respectively (Figure 4A,B).

**Figure 5:**
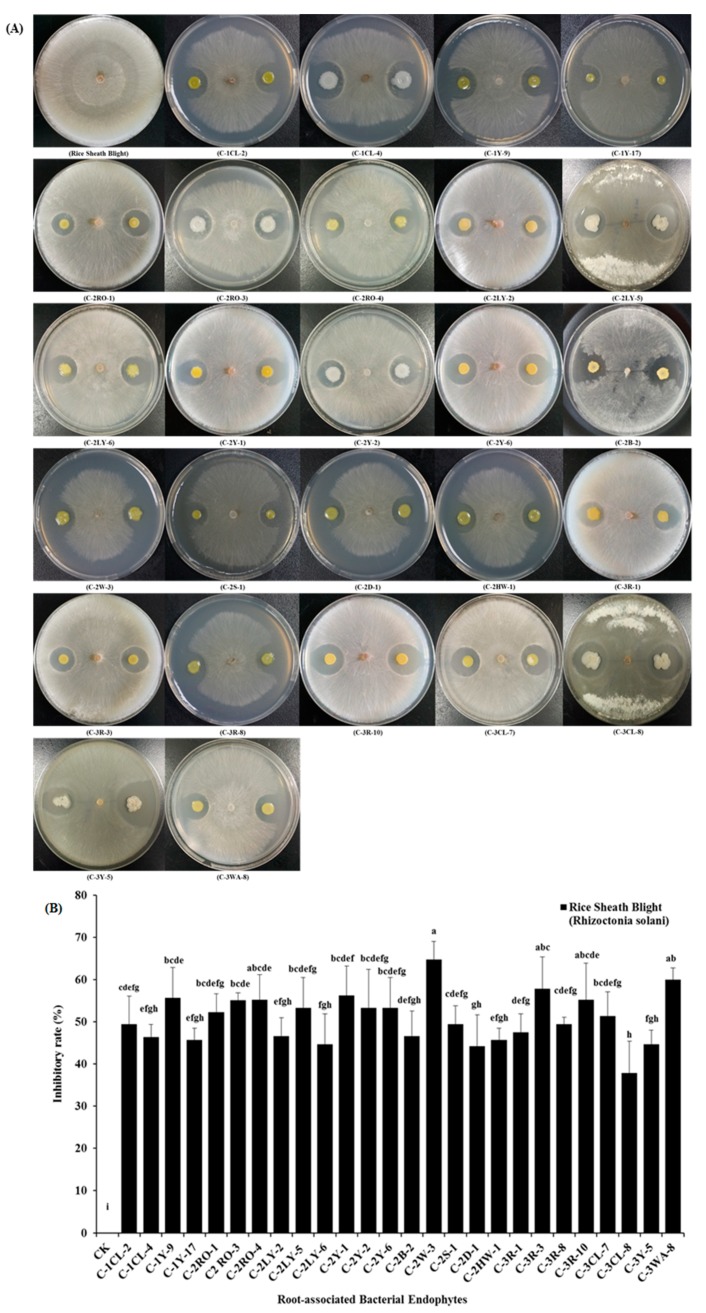
Biocontrol potential of different root-associated bacterial endophytes to rice sheath blight (*Rhizoctonia solani*), (**A**) the above plates represent in-vitro antifungal inhibitory activities by dual-culture antagonistic tests of different root-associated bacterial endophytes against rice sheath blight (*Rhizoctonia solani*), fungal phytopathogen of rice, (**B**) the above selected graphical bars indicate antifungal inhibition percentages of radial mycelial growth of (*Rhizoctonia solani*), by the 26 root-associated bacterial endophytes respectively. Values are denoted as mean ± SE (*n = 3*). Values followed by different letters are significantly different (*p* ≤ 0.05) according to the LSD test.

Additionally the photographic results explained that the 26 root-associated bacterial endophytes (i.e., C1CL-2, C1CL-4, C1Y-9, C1Y-17, C2RO-1, C2RO-3, C2RO-4, C2LY-2, C2LY-5, C2LY-6, C2Y-1, C2Y-2, C2Y-6, C2B-2, C2W-3, C2S-1, C2D-1, C2HW-1, C3R-1, C3R-3, C3R-8, C3R-10, C3CL-7, C3CL-8, C3Y-5, and C3WA-8) significantly suppressed the mycelial (*Rhizoctonia solani*) growth of rice sheath blight, with a suitable inhibitory ratio from 37.8% to 64.7%. These results demonstrated the abilities of the biocontrol potential of bacterial isolates having in-vitro dual-culture antagonistic activity. These 26 bacterial endophytes demonstrated that they were affiliated to the following bacterial species: such as *B. wiedmannii ssp., B. altitudinis ssp., B. marisflavi ssp., B. aryabhattai ssp., B. indicus ssp., B. tequilensis ssp., B. toyonensis ssp.,* and *F. phosphorivorans ssp.,* (Figure 5A,B).

After the investigation of the antagonistic activity of isolated root-associated endophytes, we observed that *B. altitudinis ssp.* and *F. phosphorivorans ssp.,* showed efficient biocontrol abilities against the tested phytopathogens, whereas *B. aryabhattai ssp.* and *B. marisflavi ssp.,* showed antifungal activities against *F. graminearum, F. moniliforme*, and *R. solani* phytopathogen species. Meanwhile, nine bacterial endophytes also expressed potential inhibitory effects against the fungal phytopathogens, such as *B. wiedmannii ssp., B. indicus ssp., B. tequilensis ssp., B. toyonensis ssp., B. cereus ssp., L. fusiformis ssp., L. mangiferihumi ssp., P. cucumis ssp.,* and *C. metallidurans ssp.,* respectively. However, the above-mentioned root-associated bacterial endophytes have not been reported previously as biocontrol agents against the four major fungal phytopathogens, i.e., *M. oryzae, F. graminearum, F. moniliforme,* and *R. solani* of rice. 

### 2.5. Molecular Detection of Antibiotic Related Genes Based on PCR Amplification of Root-Associated Bacterial Endophytes

We achieved the molecular identification of 12 different antibiotic responsive genes, including *2, 4-DAPG, PLT, PRN, PKSI, NRPS, HCN, Sfp, SrfC, ItuD, FenD, BamC*, and *Cellulase*), through the polymerase chain reaction (PCR) amplification from root-associated bacterial endophyte isolates, and the results are shown in Table S3. The (PCR) based amplification revealed that 91 bacterial endophytes were showed positive bands for *2, 4-DAPG* gene, and 110 bacterial endophytes were indicated for the positive expression of the *PLT* antibiotic gene. Furthermore, positive expression of the *PRN* gene was also verified from 85 bacterial isolates, and 90 bacterial isolates had a positive amplification of the *PKSI* gene. Along with these, a total of 102 endophytes expressed positive bands of the *NRPS* gene, and the *HCN* gene fragment was also successfully spotted in all 98 bacterial endophytes. The positive detection of the *Sfp* gene was also observed in 97 bacterial isolates. While, in 111 isolates, *SrfC* gene bands were positively expressed. In further results from the antibiotic gene amplification, the *ItuD* gene was positively detected in the 104 bacterial isolates, and *FenD* positive gene expression was observed in the presence of 104 bacterial endophytes. Similarly, *BamC* antibiotic gene-positive bands were identified in 111 of the bacterial endophytes, and *Cellulase* gene bands were observed in 94 bacterial endophytes which were amplified on the bases of the PCR detection (Table S3).

## 3. Discussion

The current demand for agricultural production requires greater attention to achieve better crop protection from the disease-causing phytopathogens. Many efforts have been made toward sustainable agricultural crop protection using environmentally-friendly biological agents [27]. The symbiotic usages of biological agents such as ‘bacterial endophytes’ within plants can promote plant growth and control diseases. The bacterial endophytes have disease controlling abilities, and have been proven to have cost-effective potential [28]. However, among the bacterial endophytes, the role of root-associated bacterial endophytes in biocontrol is rare. The role of root-associated endophytes in the biocontrol of plant phytopathogens’ diseases with the isolation of the bioactive compound, has proved to be very important [29]. The present study was done to isolate and characterize the root-associated bacterial endophytes and their biocontrol (antagonistic) effects against different fungal phytopathogens under in-vitro conditions. Similar results were reported for root-associated bacterial communities, based on their antagonistic mechanisms [30,31].

In our current investigations, about 122 root-associated bacterial endophytes were isolated from the rice plant. These endophytes were affiliated with six different bacterial genera, including *Bacillus, Fictibacillus, Lysinibacillus, Cupriavidus, Paenibacillus*, and *Microbacterium*. The analysis revealed that a *Bacillus genus* was found as a major root-associated bacterial endophyte. Furthermore, we confirmed that the 16S rRNA gene sequences-based phylogeny has been disclosed and that the above-mentioned genera have been sub-divided into 22 various types of bacterial species which represents the novelty against rice fungal pathogens (Table 1). Similar findings have previously reported 169 endophytic *actinobacteria* isolates from the various tissues of the host plant, and have identified some rare bacterial genera, such as *Amycolatopsis, Actinomycete, Brevibacterium, Kocuria, Leifsonia, Microbacterium, Micrococcus, Micromonospora, Nocardiopsis, Promicromonospora, Pseudonocardia, Rhodococcus, Saccharopolyspora,* and *Tsukamurella* [32].

Similarly, many other bacterial endophytes that promote the plant growth, yield and, suppress the pathogens, have been previously reported. For example, three phyla, *Firmicutes, Actinobacteria*, and *Proteobacteria,* from 17 different plant species have been identified. There have been 106 bacterial genera, including *Burkholderia, Bacillus, Brevundimonas, Agrobacterium, Achromobacter,* and *Acinetobacter* with 23 various types of bacterial endophytes species, found in these 17 plant species [33]. Additionally, bacterial genera, including *Herbaspirillum, Enterobacter, Cladosporium, Flavobacterium, Clavibacter, Streptomyces, Klebsiella, Stenotrophomonas, Rhodococcus, Staphylococcus, Rhizobium, Methylobactrium*, *Microbacterium*, *Microbispora*, *Paenibacillus*, *Pseudomonas*, and *Pantoea* have been studied for their specific function in the promotion of plant growth, biomass, yield, and their role as biocontrol agents [34,35,36,37,38]. Moreover, researchers have found 77 endophytic bacteria, isolated from the leaves, stems, and roots of the *Solanum nigrum* plant; 80 bacterial endophytes, identified from *Piper nigrum* L. cultivated into two indigenous environments; and 115 bacterial endophytes, from 72 varieties of plant’s leaves with the function to work against the pathogens and promoted the plant growth [39,40,41]. Similarly, in the present study, the *Bacillus* bacterial endophyte species resulted in a rice host plant with an antagonistic response against fungal phytopathogens, and it (*Bacillus*) could be suggested as a specific genus for the rice plant.

Furthermore in the current investigation, we studied that several types of biochemical and physiological characteristics, including gram reactions (GR), catalase activity (CA), endospore activity (EA), oxidase activity (OA), motility performance (MP), citrate activity (CA), gelatinase production (GP), urease activity (UA), nitrate reduction activity (NRA), Voges–Proskauer performance (VPP), methyl red activity (MRA), starch hydrolyses activity (SHA), glucose activity (GA), mannitol activity (MA), lactose activity (LA), and maltose activity (MA), from the isolated root-associated bacterial endophytes respectively (Table S2). In this study, the isolates were identified on the basis of various biochemical and physiological characteristics. These findings were supported by Passari et al. [32], who also displayed the various positive occurrences of the catalase activity, gram reaction, endospore activity, motility performance, and oxidase activities, from different endophytic *actinobacteria* isolates, having the same role as a biocontrol. 

Similarly, in the present research, we also confirmed certain other biochemical and physiological parameters from bacterial endophytes, such as citrate activity, urease activity, nitrate reduction activity, Voges–Proskauer performance, methyl red activity, and starch hydrolyses activity, glucose activity, mannitol activity, the lactose activity, and maltose activity respectively (Table S2). These results were confirmed by a few studies in past years [24,42], both researchers demonstrated the comparative results of the many biochemical and physiological activities in the positive and negative conditions such as gelatinase production, nitrate reduction, Voges–Proskauer performance, citrate activity, and starch hydrolyses activity, etc., from various bacterial endophytes with similar kind of modes of action against plant pathogens in different crops, respectively [43]. In this regard, our study relates to the root-associated bacterial endophytes in-vitro conditions to investigate the dual-culture response against rice fungal pathogens, with promising roles as biocontrol agents for sustainable agriculture and a friendly ecosystem. Further, various analyses have been defined regarding in-vitro dual-culture antagonistic tests for the screening of many bacillus endophytic bacteria as biocontrol agents against different soil and seed-born fungal pathogens such as *S. sclerotiorum, F. oxysporum, R. solani*, and *P. capsici* [5,44,45].

In this study, we discovered 10 root-associated bacterial endophytes against the rice blast disease (*Magnaporthe oryzae*) pathogen. Among the isolates, 10 (C1W-5, C1Y-10 C2RO-1, C2RO-3, C2B-2, C3R-3, C3R-10, C3Y-10, C3SP-3, and C3WA-8) bacterial endophytes had a positively antagonistic mycelial growth suppression against the phytopathogenic fungi of *M. oryzae*, with an appropriate inhibitory rate (%) under an in-vitro dual-culture environment. Also these root-associated endophytes were recognized as *B. altitudinis ssp., F. phosphorivorans ssp.,* and *C. metallidurans ssp.,* in the current study. The results are supported by Naureen et al. [46], who identified endophytic bacteria in the rhizosphere of various cereals, i.e., rice, wheat, and maize crops. These isolates were associated with the innovative genera, including *Pseudomonas, Bacillus*, and *Enterobacter* as growth promoters and as biocontrol agents under control environments.

Additionally, the results of this experiment are also supported by the results from [16], where the authors tested 11 rhizosphere bacteria against the rice blast fungal pathogen, for the development of biocontrol activities under in-vitro condition. Among these 11 rhizosphere-associated isolates, only *Pseudomonas ssp*. EA105 exhibited the 90% antagonistic effects against appressoria. Similarly, many studies also supported our results by observing the 60 bacterial isolates from the rice rhizosphere by dual-culture assay, and these isolates showed biocontrol abilities against rice blast (*M. oryzae*) pathogen [47,48,49]. Furthermore, the in-vitro antagonistic activity of various *Streptomyces spp*. isolates were also tested against the rice blast pathogen through a dual-culture test, which showed the appropriate microbial screening ability for the biocontrol activities [50].

A further 20 root-associated bacterial endophytes were antagonistically conformed against the rice seedling blight (*F. graminearum*), a fungal pathogen of rice (Figure 3A,B). These 20 root-associated bacterial endophytes are recognized as biocontrol agents against the antifungal activities of *F. graminearum*. These root-associated endophytes expressed potential abilities and suppressed mycelial growth with the highest inhibitory rate (%) in the in-vitro dual-culture antagonistic application. The response of the root-associated endophytes against the plant phytopathogens could be due to involvement in their biological mechanisms by control of the extracellular and intracellular enzymatic ribosomal compounds, such as cell divider enzymes, lipases, siderophore, hydrogen cyanide, and proteases, etc.

Moreover, in the recent past researchers have also studied similar aspects about the effective reduction of *F. graminearum* by the number of bacterial strains such as *Bacillus ssp*., *Streptomyces ssp*., and *Pseudomonas ssp*. [51,52]. Along with the above-mentioned studies, some other antifungal endophytes, such as *Cryptococcus spp*. and *Clonostachys ssp*., were reported as biocontrol agents for the head blight of wheat [53,54]. These endophytes, as biocontrol agents, have various kinds of antifungal compounds, which have an effective role against the specific pathogenic fungal growth. For example, the strain CHAO belongs to *Pseudomonas fluorescens,* which produced the siderophores enzyme, *2, 4-diacetylphloroglucinol*, and *phenazine* compounds, and showed the biocontrol performance against the *Gaeumannomyces graminis* and *Thielaviopsis basicola* fungal pathogens [55,56,57]. Further, some strains of *Bacillus spp.* were detected to have major inhibitory compounds, such as *Fengycin* and *iturin,* and represented the solid effects against the *F. graminearum* rice pathogen [55,56,57]. In addition to these, certain bacterial endophyte strains of *B. amyloliquefaciens* showed abilities to control *F. graminearum* formations in wheat crop [58], and some bacterial strains of the *P. fluorescent* EM-85 with two *Bacillus* strains MR-11 and MRF from the rhizosphere of maize, showed biocontrol potential against the *F. graminearum*, *M. phaseolina*, and *F. moniliforme* plant–fungal phytopathogens and promoted the plant growth [59].

Further, in this study, 16 root-associated bacterial endophytes were successfully screened out through expressing their biocontrol potentials against the mycelia growth of bakanae “*Fusarium moniliforme*”, a fungal phytopathogen of rice (Figure 4A,B). These 16 root-associated bacterial endophytes displayed satisfactory biocontrol capacity with a considerable range of inhibitory rate (%) against mycelial (*F. moniliforme*) growth under dual-culture antifungal antagonistic activities against. These results suggested that the root-associated endophytes showed a highly dominant response on the growth of rice pathogens, and have biocontrol potential against the *F. moniliforme*, as these endophytes have natural abilities to produce extracellular enzymatic activities, lipases formation, siderophore performances, and proteases activities. However, only a few researchers have studied the biocontrol mechanism through the bacterial endophytes for a rice seedling fungal pathogen [60,61]. Moreover, the findings of our study are supported by Chung et al. [7], who isolated a novel endophytic bacteria *B. oryzicola* YC7007 strain from the rice roots, with a strong biocontrol inhibition activity against the rice bakanae disease [7]. Along with these, some other *Bacillus ssp.* and *Pseudomonas ssp.* bacterial species are well known, with biocontrol activities in contrast to the *F. moniliforme* for the production of different antibiotics [62]. The biocontrol activities of the bacterial endophytes, including *P. cepacia, P. fluorescens,* and *B. subtilis,* have been previously identified against phytopathogenic fungi (*F. moniliforme, Pythium, Aphanomyces, R. solani, M. phaseolina, S. rolfsii,* and *F. oxysporum*), i.e., root rot of field pea [63].

The sheath blight (*R. solani*) is a major and common fungal phytopathogen of the rice plant. In this study, we have identified 26 suppressive root-associated bacterial endophytes (Figure 5A,B). These root-associated bacterial endophytes were confirmed for the detection of antifungal activities to *R. solani* with the dual-culture antagonistic test. The potential role as biocontrol agents of the 26 root-related bacterial endophytes was studied for the first time against the rice sheath blight disease (Figure 5A,B). Similarly, about 576 bacterial endophytes from the different rice cultivars showed high antagonistic effects against *R. solani* and *F. oxysporum* growth under in-vitro conditions and they improve the rice plant growth. Most of these isolates were belong to bacterial genera such as *Paenibacillus spp., Microbacterium spp., Bacillus spp.,* and *Klebsiella spp*. [64]. Furthermore, Smitha et al. [65] investigated the antifungal effect by the bacterial strain of *B. amyloliquefaciens*-SN13 against *R. solani* under in-vitro conditions. Another researcher also investigated that the *Bacillus subtilis* 330-2 strain, which expressed antagonistically biocontrol activities with a good mycelial inhibition against the different fungal phytopathogens, i.e., *R solani* AG1, *N. oryzae*, *C. heterostrophus*, *F. oxysporum B. cinerea*, and *A. alternate,* under in-vitro conditions [66]. Additionally, in the various previously reported bacterial endophytes, isolates from different crops have been applied antagonistically for the biocontrol aspects, and these endophytes showed very solid inhibitory antifungal activities against the different kind of fungal phytopathogens, including some endophytes that displayed antifungal activities against *R. solani* under in-vitro environments [67,68].

The molecular identification of antibiotic-related genes (*2, 4-DAPG, PLT, PRN, PKSI, NRPS, HCN, Sfp, SrfC, ItuD, FenD, BamC,* and *Cellulase*) in the isolated bacterial endophytes, which involved in various biocontrol activities, were assessed by PCR-based amplification (Table S3). In the present study, after the amplification, we found that the majority of root-associated bacterial endophytes clearly displayed a positive indication regarding the 12 antibiotic-related genes. The findings of this study were supported by Kushwaha et al. [24]. They described the PCR based amplification of antibiotic peptide related genes, including *ItuD, BmyC,* and *srfA,* from the 19 *Bacillus* endophytic bacterial strains with their biocontrol character in pearl millet cereal crop [24]. Moreover, Xu et al. (2019) also published similar results related to functional biosynthesis antibiotic-related genes, such as *PKSI*, *NRPS*, *ItuD*, *SrfC*, and *Sfp,* by PCR from 33 different endophytic bacterial species, where 26 isolates from *Bacillus ssp*., showed antifungal activities against different phytopathogens of the mulberry plant respectively [25]. Additionally, the *PKSI*, *PKSII,* and *NRPS,* genes involved in the production of a secondary metabolite against the fungal and bacterial phytopathogens, have been identified in 81 different endophytic *actinobacterial* species, that were isolated from the *Rhynchotoechum ellipticum* plant [32]. Further, many positive biocontrol encoding gene traits, such as *Pyrrolnitrin*, *Pyoluteorin*, *2, 4-diacetylphloroglucinol, Bacillomycin D, Hydrogen cyanide, Fengycin, Cellulase,* and *Lytic enzyme* from 16 the rhizobacterial isolates, were discovered using several gene-specific primers, which were strongly associated to *Bacillus* and *Pseudomonas* bacterial species respectively [14].

The main findings of this study revealed that *Bacillus* bacterial endophyte species were found in rice host plants with an antagonistic response against fungal phytopathogens, and that *Bacillus* could be suggested as a specific genus to check the biocontrol response against rice fungal pathogens. Further, this study proposed the effectiveness of PCR based identifications of the antibiotic-related genes, which produce a range of efficient bioactive compounds that are useful to understand the mechanism of antifungal activities of bacterial endophytes against the phytopathogens.

## 4. Materials and Methods 

### 4.1. Plant Material and Sampling Location

The present research was conducted on the isolation of endophytic bacterial strains from rice roots. Samples for the isolation of the bacterial strain were collected from rice cultivars (Xiushui-48, Y-003, and CO-39) grown in the field. The root samples were collected randomly every 5 meters, from the rice demonstration field of China National Rice Research Institute, (CNRRI), Hangzhou, China. The carefully collected samples were put into sterile polythene bags with proper tagging, kept in darkness to save them from environmental stresses, and then immediately brought to the laboratory in an icebox for surface sterilization.

### 4.2. Surface Sterilization of Rice Root Tissues

Randomly collected root samples from rice plants were washed with tap water to clean and remove the soil particles. From the separated clean root samples, about 15 g were taken and cut into 2–3 cm fine pieces with a sterile surgical blade. Then these small pieces were put on sterile Petri plates for initial sterilization and later the samples were again rewashed with sterilized double-distilled water (ddH_2_O) ten times with 2–4 min intervals. These surface-sterilized root samples were immersed in 70% ethanol solution for 4 min, and then these samples were sterilized with sodium hypochlorite solution (2.5% chlorine) for 5 min followed by washing three times with ddH_2_O. Finally, these root samples were washed ten times with ddH_2_O for 4–5 min to remove the remaining excessive sodium hypochlorite contents. The final sterilized root samples were crushed by autoclaved pestle and mortar to obtain the extract, under the laminar airflow hood (Suzhou Antai Air Technology Co., Ltd., Suzhou, China). From the extract, the 5µl solution was taken and placed on Luria–Brentani (LB) solid agar medium plates with three replications. The inoculated LB plates were incubated at 30 ± 2 °C in a growth incubator for 24–72 hours for observation of the initial growth of root-associated bacterial colonies [69].

### 4.3. Isolation of Root-Associated Bacterial Endophytes

After the confirmation of the initial growth observations of root-associated bacterial endophytic colonies, the single bacterial colonies based on morphological characteristics were re-streaked on LB agar plates, and the samples were incubated at 30 ± 2 °C for 24 hours in the dark, for the culture purification of root-associated bacterial endophytic strains. Once the bacterial isolates were purified, the single bacterial colonies from each of the purified plate samples were inoculated separately into separately 12 mL Eppendorf tubes containing 2 mL liquid LB medium. These inoculating bacterial tubes were grown overnight in a shaking incubator, at 28 ± 2 °C with 200 rounds per min (rpm) in dark conditions. After the overnight incubation, 1 ml of each endophytic bacterial strain were transferred into 2 mL pre-sterilized Eppendorf tubes containing an equal quantity (1 mL) of 40% (v/v) glycerol, and all isolated endophytic strains were preserved at −80 °C for further use. Further, 1 mL bacterial colonies were used for the DNA extraction and amplification of 16S rRNA genes [70].

### 4.4. Biochemical and Physiological Characterization of Root-Associated Bacterial Endophytes

The classification of biochemical properties, including; gram reaction (GR), catalase activity (CA), endospore activity (EA), oxidase activity (OA), motility performance (MP), citrate activity (CA), and gelatinase production (GP) were detected from each of the root-associated bacterial endophytes followed by Kushwaha et al. [24]. Determination of the physiological traits, such as; starch hydrolyses activity (SHA), nitrate reduction activity (NRA), Voges–Proskauer performance (VPP), methyl red activity (MRA), glucose activity (GA), mannitol activity (MA), lactose activity (LA), and maltose activity (MA) of all the bacterial endophytes were characterized by following Islam et al. [42]. All analysis was repeated three times and respective results of all tests are mentioned with positive and negative symbols (Table S2).

### 4.5. Genomic DNA Extraction of Root-Associated Bacterial Endophytes

The genomic DNA extraction from root-associated bacterial endophytes was performed using the DNAiso Reagent Kit (TaKaRa Biotechnology, Co., Ltd, Dalian, China) according to the manufacturer’s instructions. The quantity and quality of genomic DNA were observed by the Nano Drop 2000c Spectrophotometer (Thermo Fisher Scientific, Waltham, MA, USA) at the 260 nm and 280 nm. The purity of the extracted bacterial genomic DNA samples was further assessed through the 1% agarose gel electrophoresis with a 1 x TAX buffer solution, following the described procedure of Koh et al. [71].

### 4.6. PCR Amplification and Classification of Root-Associated Bacterial Endophytes 

The total extracted DNA from each isolate was used for 16S rRNA gene amplification using the Sanger sequencing method from (Sangon Biotechnology Co., Ltd., Shanghai, China). The PCR amplification was performed by using a 50 µL mixture reaction and the 16S rRNA gene was amplified through two universal bacterial primer sets, 27F (5’-AGAGTTTGATCTTGGCTCAG-3’) and 1492R (5’TACGGTTACCTTGTTACGACTT- 3’). The 50 µL PCR mixture reaction contains 1 µL of DNA template (50 ng genomic DNA), 5.0 µL (10 x PCR reaction buffer), 4.0 µL of dNTPs (2.5 mM) mixture, 0.5 µL rTaq DNA polymerase (Thermo Fisher Scientific, Waltham, USA), 0.75 µL from each of 27F and 1492R primer (20 mM), and the remaining volume adjusted up to 50 µL with autoclaved ultra-pure distilled water (ddH_2_O) with some modifications [72]. The 16S rRNA amplification was performed in a Bioer xp PCR thermal cycler with the following conditions: the initial denaturation was done at 94 °C for 5 min, followed by 30 amplification cycles, annealing at 55 °C for the 30 s, an extension at 72 °C for 90 s, and the final extension step at 72 °C for 5 min. The amplified DNA products were loaded with 6× DNA loading buffer (Thermo Fisher Scientific, Waltham, USA) in 1% agarose gel (w/v) using in 1x Tris-acetate EDTA (TAE) buffer solution for one hour at 100 V electrophoresis. The agarose gel was stained with GelRed (Biotium Inc.) and the PCR product bands were examined under UV light in a UV transilluminator (Bio-Red Molecular Imager Gel Doc XR+ system). Whereas the DNA band gel image was captured with Image Lab Software. Furthermore, the remaining amplified 16S rRNA gene products were sent to the sequencing to the commercial laboratory (TaKaRa Biotechnology Co., Ltd, Dalian, China) for sequencing. The 16S rRNA gene sequences of the bacterial isolates were assembled and matched by EzBioCloud (www.ezbiocloud.net), and also searched against the National Centre for Biotechnology Information (NCBI) database to identify the corresponding homologous sequences for this using Basic Local Alignment Search Tool (BLAST) (http://www.ncbi.nlm.nih.Gov/BLAST).

### 4.7. 16S rRNA Gene Sequences Based Phylogeny Analysis of Root-Associated Bacterial Endophytes 

The 16S rRNA gene sequences-based phylogeny analysis was performed on the bases of 16S rRNA gene sequences and established that the taxonomic position of the bacterial endophytes was aligned, using the MUSCLE alignment through the neighbor-joining method, by the most closely related bacterial type strains [73]. The 16S rRNA gene sequences-based tree was constructed using MEGA 7 software [74] with the bootstrap values for 1000 replications [75].

### 4.8. Rice Fungal Phytopathogens and Culture Condition

The four rice fungal phytopathogenic diseases, rice blast (*M. oryzae*), rice sheath blight (*R.solani*), rice bakanae disease (*F. moniliforme*), and seedling blight of rice (*F. graminearum*), were preserved in the research laboratory. All disease-causing fungal pathogens were obtained from the respective laboratory culture collections, and disease mycelia were initially grown on the potato dextrose agar (PDA) medium with the following composition (potato-200 g/L, D-glucose-20 g/L, and agar-15 g/L), and the medium was autoclaved at the 121 °C for 20 min. The initially important rice pathogens were inoculated on PDA plates and these plates were incubated at 28 °C for 7–10 days for better mycelia development according to the disease growth habit (DGH), and rice pathogens were cultured three to five times, following Bibi et al. [70] with little modification. After the 7–10 days of initial growth incubation, a 5 mm diameter mycelial disc was picked from every fungal pathogenic disease and then the 5 mm disc was again placed on freshly prepared PDA plates. These plates were further incubated at 28 °C for 7–10 days. The dual-culture antagonistic activities of the entire root-associated bacterial strains were determined through measuring the inhibitory zones of mycelial development on the fungal pathogens from the PDA medium plates under in-vitro conditions. Every single clone of root-associated bacterial strains was picked up from the per-culture stoke and was then inoculated in 2 mL liquid LB medium, and the inoculated tubs were placed in shaking incubator overnight at 28 °C and 200 rpm. Further, 3 µL from each overnight amplified bacterial culture was stacked twice on the PDA plates under in-vitro conditions, and 3 µL of culture was inoculated on the fresh prepared PDA medium at two opposite side corners with an equal distance of 3 cm from the center. Finally, the inoculated plates were incubated at 28 ± 1 °C for 24 hours. After the 24-hour incubation, the freshly grown fungal mycelium was applied from fungal plates and 5 mm diameter plug discs were placed at the center of PDA plates. After that, the plates were sealed with the para-film under the dark condition and incubated at 28 °C which is the ideal requirement of the pathogenic diseases’ developments. The inhibitory zone of grown fungal pathogens was determined by recording the diameter of the inhibition area (cm). The rate of inhibition percentage was calculated with the following formula, as reported by Shakeel et al. [76].

(1) Inhibition rate (%)=(C−T)∗100/C

where “C” is the fungal pathogen mycelium diameter growth (cm) on the control plates (without bacterial inoculation) and “T” is the fungal pathogen mycelium growth diameter (cm) in the inoculated bacterial plates. The experiment was performed with three biological replications.

### 4.9. Molecular Detection of Antibiotic Related Genes Based on PCR Amplification of Root-Associated Bacterial Endophytes

The total genomic DNA of all bacterial endophytes was extracted using the DNAiso Reagent Kit (TaKaRa Biotechnology Co., Ltd, Dalian, China), according to the manufacturer’s instructions. The isolated DNA of each endophytes was used to detect the number of genes related to antibiotic biosynthesis, such as 2, 4-diacetyphloroglucinol (*2, 4-DAPG*), pyoluteorin (*PLT*), pyrrolnitrin (*PRN*), polyketide synthase (*PKSI*), non-ribosomal peptide synthetases (*NRPS*), hydrogen cyanide (*HCN*), surfactin biosynthesis (*Sfp*), surfactin synthase (*SrfC*), iturin A biosynthesis (*ItuD*), fengycin biosynthesis (*FenD*), bacillomycin D (*BamC*), and *cellulase*. To investigate the putative antifungal mechanism by the direct antagonism, and the genes associated with the production of antibiotic biosynthesis, substances were determined by using PCR amplification. The specific primers related to antibiotic genes were synthesized according to the selected sequences from the encoding region of all functional genes, as described by [14,25]. The details for the PCR amplification of all functional genes, along with the primers’ information, are presented in Table S1. The PCR amplifications were performed in a 25 µL reaction mixture, and the reaction contains 2 µL (50 ng) of DNA template, 2.5 µL of 10 x PCR reaction buffer (Mg^2+^ plus), 2 µL (2.5 mM) of dNTPs, 0.5 µL of rTaq DNA polymerase, 1 µL of each (20 mM) primers, as well as (ddH_2_O) to reach up to 25 µL. The reactions were individually operated by automatic Bio-Rad Thermal Cycler (T100^™^) respectively.

### 4.10. Statistical Analysis 

All rice root-endophytes were tested against the rice fungal pathogens. The rate of inhibition zone was performed with three biological replications. The whole experiment data were statistically analyzed by one-way analysis of variance (ANOVA) using the statistics software—8.1 version (Analytical Software, Tallahassee, FL, USA). The mean-variance comparison was analyzed by using a Fisher protected least significant difference (LSD) test at the 0.05 probability level. The values were expressed as means ± SE of the successfully inhibited endophytic isolates to phytopathogens.

## 5. Conclusions

We concluded that 122 root-associated bacterial endophytes in rice plant were obtained with a diversity of physiological and biochemical characteristics against fungal phytopathogens. According to the 16S rRNA gene sequencings and its phylogeny analysis, the results showed that *B. altitudinis ssp.* and *B. aryabhattai ssp.* were found as the predominant endophytes. Additionally, 71 root-associated bacterial endophytes successfully presented antagonistic activities against the *M. oryzae*, *F. graminearum*, *F. moniliforme*, and *R. solani* fungal phytopathogens of rice. Furthermore, we confirmed that most of the bacterial endophytes exhibited positive growth in biochemical and physiological properties, and that the majority of bacterial endophytes indicated a strong ability with different biocontrol traits, which is responsible for the antifungal activities through biosynthesis of encoding antibiotic responsive genes. The current experimental findings were highly encouraging for the theory that most of the root-associated bacterial endophytes have significant biocontrol potential against the major fungal phytopathogens with antagonistic effects. These bacterial endophytes could be considered beneficial for dual purposes, including plant growth-promoting activities, development of biopesticides, and biofertilizers for sustainable agriculture and environment safety. Moreover, the outcomes of this research identify new bacterial groups as biocontrol agents with a better capacity to defend the rice from fungal pathogenic diseases for sustainable agriculture and environmental safety.

## Figures and Tables

**Table 1:** The molecular characterizations of root-associated bacterial endophytes isolated from different rice cultivar roots by 16S rRNA gene sequencings.

Associated Cultivars	Endophyte Bacterial ID Code ^(a)^	Amplified 16S rRNA Gene (bps) ^(b)^	NCBI Accession No 16S rRNA Gene Sequences ^(c)^	Closely Related Species, NCBI Accession No 16S rRNA Gene Sequence ^(d)^	Similarity (%)	Completeness (%)
Xiushui-48	C-1B-2	1418	MN543754	*Bacillus marisflavi* JCM11544^(T)^ LGUE01000011	99.8	96.5
Xiushui-48	C-1B-3	1413	MN543755	*Bacillus altitudinis* 41KF2b^(T)^ASJC01000029	99.7	96.3
Xiushui-48	C-1B-5	1412	MN543756	*Lysinibacillus fusiformis* NBRC15717^(T)^ AB271743	99.6	96.1
Xiushui-48	C-1B-6	1412	MN543757	*Bacillus cereus* ATCC14579^(T)^ AE016877	99.9	96.1
Xiushui-48	C-1B-7	1412	MN543758	*Bacillus cereus* ATCC14579^(T)^ AE016877	100	96.4
Xiushui-48	C-1B-9	1392	MN543759	*Bacillus aryabhattai* B8W22^(T)^ EF114313	99.9	94.6
Xiushui-48	C-1CL-2	1392	MN543760	*Bacillus wiedmannii* FSLW8-0169^(T)^ LOBC01000053	100	94.6
Xiushui-48	C-1CL-4	1372	MN543761	*Bacillus wiedmannii* FSLW8-0169^(T)^ LOBC01000053	100	93.1
Xiushui-48	C-1F-2	1396	MN543762	*Bacillus pseudomycoides* DSM12442^(T)^ ACMX01000133	99.8	95.1
Xiushui-48	C-1F-3	1374	MN543763	*Bacillus wiedmannii* FSLW8-0169^(T)^ LOBC01000053	100	93.3
Xiushui-48	C-1G-1	1355	MN543764	*Bacillus thioparans* BMP-1^(T)^ DQ371431	99.4	92.1
Xiushui-48	C-1G-3	1413	MN543765	*Bacillus altitudinis* 41KF2b^(T)^ ASJC01000029	99.7	96.3
Xiushui-48	C-1S-1	1379	MN543766	*Bacillus subterraneous* DSM13966^(T)^ FR733689	99.4	93.5
Xiushui-48	C-1S-2	1376	MN543767	*Bacillus lehensis* MLB-2^(T)^ AY793550	99.9	93.7
Xiushui-48	C-1S-3	1409	MN543768	*Fictibacillus phosphorivorans* Ca7T^(T)^ JX258924	99.9	96.1
Xiushui-48	C-1S-4	1379	MN543769	*Bacillus subterraneous* DSM13966^(T)^ FR733689	99.4	93.6
Xiushui-48	C-1S-5	1412	MN543770	*Bacillus acidiceler* CBD119^(T)^ DQ374637	99.7	96.2
Xiushui-48	C-1ST-4	1375	MN543771	*Bacillus subterraneous* DSM13966^(T)^ FR733689	99.3	93.5
Xiushui-48	C-1ST-5	1355	MN543772	*Bacillus thioparans* BMP-1^(T)^ DQ371431	99.8	92.1
Xiushui-48	C-1W-3	1388	MN543773	*Lysinibacillus fusiformis* NBRC15717^(T)^ AB271743	99.7	94.2
Xiushui-48	C-1W-5	1393	MN543774	*Bacillus altitudinis* 41KF2b^(T)^ ASJC01000029	99.9	94.7
Xiushui-48	C-1W-6	1393	MN543775	*Bacillus altitudinis* 41KF2b^(T)^ ASJC01000029	99.8	95.0
Xiushui-48	C-1W-14	1391	MN543776	*Bacillus altitudinis* 41KF2b^(T)^ ASJC01000029	99.8	94.6
Xiushui-48	C-1WF-1	1382	MN543777	*Bacillus aryabhattai* B8W22^(T)^ EF114313	99.8	93.9
Xiushui-48	C-1WF-2	1413	MN543778	*Bacillus altitudinis* 41KF2b^(T)^ ASJC01000029	99.7	96.3
Xiushui-48	C-1WF-3	1403	MN543779	*Lysinibacillus fusiformis* NBRC15717^(T)^ AB271743	99.7	95.5
Xiushui-48	C-1WF-7	1401	MN543780	*Bacillus aryabhattai* B8W22^(T)^ EF114313	100	95.3
Xiushui-48	C-1WF-9	1389	MN543781	*Bacillus aryabhattai* B8W22^(T)^ EF114313	100	94.4
Xiushui-48	C-1WNF-1	1376	MN543782	*Bacillus aryabhattai* B8W22^(T)^ EF114313	100	93.5
Xiushui-48	C-1WNF-2	1392	MN543783	*Bacillus aryabhattai* B8W22^(T)^ EF114313	99.9	94.6
Xiushui-48	C-1WNF-7	1412	MN543784	*Bacillus altitudinis* 41KF2b^(T)^ ASJC01000029	99.7	96.3
Xiushui-48	C-1WNF-8	1422	MN543785	*Bacillus aryabhattai* B8W22^(T)^ EF114313	99.5	96.9
Xiushui-48	C-1WNF-9	1408	MN543786	*Bacillus aryabhattai* B8W22^(T)^ EF114313	99.9	95.7
Xiushui-48	C-1Y-1	1407	MN543787	*Fictibacillus phosphorivorans* Ca7T^(T)^ JX258924	99.9	95.9
Xiushui-48	C-1Y-2	1412	MN543788	*Fictibacillus nanhaiensis* JSM082006^(T)^ GU477780	99.8	96.3
Xiushui-48	C-1Y-4	1388	MN543789	*Bacillus marisflavi* JCM11544^(T)^ LGUE01000011	99.9	94.2
Xiushui-48	C-1Y-7	1422	MN543790	*Fictibacillus nanhaiensis* JSM082006^(T)^ GU477780	99.8	96.3
Xiushui-48	C-1Y-8	1413	MN543791	*Fictibacillus phosphorivorans* Ca7T^(T)^ JX258924	99.8	96.3
Xiushui-48	C-1Y-9	1371	MN543792	*Fictibacillus phosphorivorans* Ca7T^(T)^ JX258924	99.9	93.3
Xiushui-48	C-1Y-10	1391	MN543793	*Fictibacillus phosphorivorans* Ca7T^(T)^ JX258924	100	94.6
Xiushui-48	C-1Y-11	1418	MN543794	*Fictibacillus nanhaiensis* JSM082006^(T)^ GU477780	99.9	96.6
Xiushui-48	C-1Y-12	1412	MN543795	*Fictibacillus nanhaiensis* JSM082006^(T)^ GU477780	100	95.8
Xiushui-48	C-1Y-13	1390	MN543796	*Fictibacillus phosphorivorans* Ca7T^(T)^ JX258924	100	94.6
Xiushui-48	C-1Y-16	1413	MN543797	*Fictibacillus phosphorivorans* Ca7T^(T)^ JX258924	99.8	96.3
Xiushui-48	C-1Y-17	1404	MN543798	*Fictibacillus phosphorivorans* Ca7T^(T)^ JX258924	99.9	95.7
Y-003	C-2B-1	1424	MN543799	*Paenibacillus alvei* NBRC3343^(T)^ NR113577.1	99.2	99.0
Y-003	C-2B-2	1407	MN543800	*Bacillus altitudinis* 41KF2b^(T)^ ASJC01000029	100	95.9
Y-003	C-2CL-1	1390	MN543801	*Bacillus aryabhattai* B8W22^(T)^ EF114313	99.9	94.5
Y-003	C-2D-1	1411	MN543802	*Bacillus altitudinis* 41KF2b^(T)^ ASJC01000029	99.5	96.3
Y-003	C-2GL-1	1428	MN543803	*Lysinibacillus fusiformis* NBRC15717^(T)^ AB271743	99.3	97.1
Y-003	C-2GL-3	1425	MN543804	*Lysinibacillus fusiformis* NBRC15717^(T)^ AB271743	99.7	96.7
Y-003	C-2HW-1	1390	MN543805	*Bacillus aryabhattai* B8W22^(T)^ EF114313	99.9	100
Y-003	C-2HW-2	1390	MN543806	*Bacillus cereus* ATCC 14579^(T)^ AE016877	99.8	94.5
Y-003	C-2HW-3	1430	MN543807	*Lysinibacillus mangiferihumi* M-GX18^(T)^ JF731238	99.9	97.1
Y-003	C-2HW-4	1372	MN543808	*Bacillus aryabhattai* B8W22^(T)^ EF114313	100	93.2
Y-003	C-2HW-5	1328	MN543809	*Bacillus aryabhattai* B8W22^(T)^ EF114313	99.9	94.0
Y-003	C-2HW-6	1425	MN543810	*Bacillus altitudinis* 41KF2b^(T)^ ASJC01000029	99.7	96.9
Y-003	C-2HW-7	1429	MN543811	*Paenibacillus alvei* NBRC3343^(T)^ NR113577.1	99.2	99.0
Y-003	C-2LY-1	1404	MN543812	*Bacillus aryabhattai* B8W22^(T)^ EF114313	100	95.5
Y-003	C-2LY-2	1394	MN543813	*Bacillus marisflavi* JCM11544^(T)^ LGUE01000011	100	94.8
Y-003	C-2LY-4	1380	MN543814	*Lysinibacillus fusiformis* NBRC15717^(T)^ AB271743	99.7	93.7
Y-003	C-2LY-5	1379	MN543815	*Bacillus marisflavi* JCM11544^(T)^ LGUE01000011	99.9	93.7
Y-003	C-2LY-6	1388	MN543816	*Bacillus aryabhattai* B8W22^(T)^ EF114313	100	94.4
Y-003	C-2LY-7	1372	MN543817	*Bacillus aryabhattai* B8W22^(T)^ EF114313	100	93.2
Y-003	C-2R-1	1406	MN543818	*Bacillus altitudinis* 41KF2b^(T)^ ASJC01000029	100	95.9
Y-003	C-2RO-1	1417	MN543819	*Bacillus altitudinis* 41KF2b^(T)^ ASJC01000029	100	96.5
Y-003	C-2RO-3	1403	MN543820	*Bacillus altitudinis* 41KF2b^(T)^ ASJC01000029	100	95.4
Y-003	C-2RO-4	1416	MN543821	*Bacillus altitudinis* 41KF2b^(T)^ ASJC01000029	99.6	96.7
Y-003	C-2S-1	1391	MN543822	*Bacillus altitudinis* 41KF2b^(T)^ ASJC01000029	99.7	94.8
Y-003	C-2SN-1	1393	MN543823	*Bacillus cereus* ATCC14579^(T)^ AE016877	100	94.7
Y-003	C-2SN-2	1410	MN543824	*Bacillus altitudinis* 41KF2b^(T)^ ASJC01000029	99.7	96.0
Y-003	C-2SN-3	1410	MN543825	*Bacillus altitudinis* 41KF2b^(T)^ ASJC01000029	99.6	96.1
Y-003	C-2SP-1	1410	MN543826	*Bacillus altitudinis* 41KF2b^(T)^ ASJC01000029	99.6	96.3
Y-003	C-2T-1	1375	MN543827	*Paenibacillus cucumis* AP-115^(T)^ KU201962	99.5	93.0
Y-003	C-2W-1	1406	MN543828	*Bacillus cereus* ATCC14579^(T)^ AE016877	100	95.4
Y-003	C-2W-2	1374	MN543829	*Bacillus cereus* ATCC14579^(T)^ AE016877	100	93.3
Y-003	C-2W-3	1426	MN543830	*Bacillus tequilensis* KCTC13622^(T)^ AYTO01000043	99.7	96.9
Y-003	C-2W-4	1421	MN543831	*Bacillus cereus* ATCC14579^(T)^ AE016877	100	96.7
Y-003	C-2W-5	1420	MN543832	*Bacillus cereus* ATCC14579^(T)^ AE016877	100	100
Y-003	C-2Y-1	1379	MN543833	*Bacillus indicus* LMG22858^(T)^ JGVU01000003	99.9	94.7
Y-003	C-2Y-2	1403	MN543834	*Bacillus marisflavi* JCM11544^(T)^ LGUE01000011	100	95.5
Y-003	C-2Y-3	1424	MN543835	*Bacillus altitudinis* 41KF2b^(T)^ ASJC01000029	99.8	96.9
Y-003	C-2Y-4	1411	MN543836	*Bacillus altitudinis* 41KF2b^(T)^ ASJC01000029	99.8	96.3
Y-003	C-2Y-5	1429	MN543837	*Bacillus cereus* ATCC14579^(T)^ AE016877	99.7	97.0
Y-003	C-2Y-6	1394	MN543838	*Bacillus marisflavi* JCM11544^(T)^ LGUE01000011	100	94.8
CO-39	C-3CL-1	1402	MN543839	*Bacillus wiedmannii* FSLW80169^(T)^ LOBC01000053	100	95.3
CO-39	C-3CL-3	1394	MN543840	*Bacillus wiedmannii* FSLW80169^(T)^ LOBC01000053	100	94.8
CO-39	C-3CL-4	1383	MN543841	*Bacillus wiedmannii* FSLW80169^(T)^ LOBC01000053	100	93.9
CO-39	C-3CL-5	1445	MN543842	*Bacillus cereus* ATCC14579^(T)^ AE016877	99.7	98.4
CO-39	C-3CL-6	1430	MN543843	*Bacillus cereus* ATCC14579^(T)^ AE016877	100	97.1
CO-39	C-3CL-7	1445	MN543844	*Bacillus tequilensis* KCTC13622^(T)^ AYTO01000043	99.7	96.9
CO-39	C-3CL-8	1418	MN543845	*Bacillus wiedmannii* FSLW80169^(T)^ LOBC01000053	99.9	96.7
CO-39	C-3F-5	1438	MN543846	*Lysinibacillus mangiferihumi* MGX18^(T)^ JF731238	99.2	97.6
CO-39	C-3F-7	1429	MN543847	*Bacillus altitudinis* 41KF2b^(T)^ ASJC01000029	99.7	97.1
CO-39	C-3F-8	1439	MN543848	*Lysinibacillus fusiformis* NBRC15717^(T)^ AB271743	99.6	97.9
CO-39	C-3G-1	1423	MN543849	*Bacillus wiedmannii* FSLW80169^(T)^ LOBC01000053	99.7	96.9
CO-39	C-3P-3	1380	MN543850	*Cupriavidus metallidurans* CH34^(T)^ CP000353	99.6	95.2
CO-39	C-3P-5	1408	MN543851	*Cupriavidus metallidurans* CH34^(T)^ CP000353	99.2	97.2
CO-39	C-3R-1	1416	MN543852	*Bacillus altitudinis* 41KF2b^(T)^ ASJC01000029	100	96.3
CO-39	C-3R-2	1428	MN543853	*Bacillus aryabhattai* B8W22^(T)^ EF114313	99.3	96.9
CO-39	C-3R-3	1427	MN543854	*Bacillus altitudinis* 41KF2b^(T)^ ASJC01000029	99.9	97.1
CO-39	C-3R-5	1407	MN543855	*Bacillus altitudinis* 41KF2b^(T)^ ASJC01000029	99.7	96.0
CO-39	C-3R-6	1414	MN543856	*Bacillus altitudinis* 41KF2b^(T)^ ASJC01000029	99.7	96.5
CO-39	C-3R-7	1418	MN543857	*Lysinibacillus fusiformis* NBRC15717^(T)^ AB271743	99.5	96.5
CO-39	C-3R-8	1437	MN543858	*Bacillus altitudinis* 41KF2b^(T)^ ASJC01000029	99.9	98.0
CO-39	C-3R-9	1382	MN543859	*Bacillus cereus* ATCC14579^(T)^ AE016877	100	93.8
CO-39	C-3R-10	1425	MN543860	*Bacillus altitudinis* 41KF2b^(T)^ ASJC01000029	100	96.9
CO-39	C-3SP-3	1399	MN543861	*Cupriavidus metallidurans* CH34^(T)^ CP000353	100	93.3
CO-39	C-3T-1	1355	MN543862	*Cupriavidus metallidurans* CH34^(T)^ CP000353	100	93.3
CO-39	C-3T-2	1380	MN543863	*Cupriavidus metallidurans* CH34^(T)^ CP000353	99.6	95.2
CO-39	C-3T-3	1398	MN543864	*Cupriavidus metallidurans* CH34^(T)^ CP000353	99.6	96.4
CO-39	C-3T-4	1413	MN543865	*Paenibacillus uliginis* N3/975^(T)^ NR117012.1	96.6	99.0
CO-39	C-3T-5	1364	MN543866	*Cupriavidus metallidurans* CH34^(T)^ CP000353	99.2	97.3
CO-39	C-3T-6	1407	MN543867	*Cupriavidus metallidurans* CH34^(T)^ CP000353	99.5	94.0
CO-39	C-3T-7	1399	MN543868	*Paenibacillus cucumis* AP-115^(T)^ KU201962	99.6	94.9
CO-39	C-3T-8	1399	MN543869	*Cupriavidus metallidurans* CH34^(T)^ CP000353	99.5	96.6
CO-39	C-3T-9	1355	MN543870	*Microbacterium laevaniformans* DSM20140^(T)^ Y17234	99.7	93.8
CO-39	C-3W-3	1418	MN543871	*Lysinibacillus fusiformis* NBRC15717^(T)^ AB271743	99.4	96.6
CO-39	C-3WA-8	1395	MN543872	*Bacillus altitudinis* 41KF2b^(T)^ ASJC01000029	99.8	95.0
CO-39	C-3Y-2	1415	MN543873	*Bacillus marisflavi* JCM11544^(T)^ LGUE01000011	99.9	96.5
CO-39	C-3Y-5	1416	MN543874	*Bacillus marisflavi* JCM11544^(T)^ LGUE01000011	100	96.6
CO-39	C-3Y-10	1390	MN543875	*Fictibacillus phosphorivorans* Ca7^(T)^ JX258924	100	94.6

**Note**: (**a**) C1, C2, and C3, indicates cultivar numbers; 1, 2, and 3, and different alphabetical letters indicate various bacterial morphological codes during isolation, **(b)** represented the 16S rRNA gene sequence base pairs (bps) used in this study, **(c)** shows the NCBI GenBank accession numbers of current research **(d)** indicated by the closely related species, NCBI bank accession no of 16S rRNA gene sequence as reference root-associated bacterial endophytes species.

## Supplementary Materials

The following are available online at https://www.mdpi.com/2076-0817/9/3/172/s1, Table S1: List of used primers in the present study for detection of biosynthesis antibiotic-related genes, from potential root-associated bacterial endophytes, by PCR amplifications, Table S2: Identifications of biochemical and physiological characteristics of root-associated bacterial endophytes derived from rice host, Table S3: Molecularly determination of functional biosynthesis antibiotic-related genes from root-associated bacterial endophytes.

## Abbreviations

PCR	=	Polymer Chain Reaction
C-1	=	Cultivar One (Xiushui-48)
C-2	=	Cultivar Two (Y-003)
C-3	=	Cultivar Three (CO-39)
CNRRI	=	China National Rice Research Institute
LB	=	Luria Bertani
PDA	=	Potato Dextrose Agar
RPM	=	Rounds Per Mint
DGH	=	Disease Growth Habit
TAE	=	Tris-acetate EDTA
GR	=	Gram Reaction
CA	=	Catalase Activity
EA	=	Endospore Activity
OA	=	Oxidase Activity
MP	=	Motility Performance
CA	=	Citrate Activity
GP	=	Gelatinase Production
SHA	=	Starch Hydrolyses Activity
NRA	=	Nitrate Reduction Activity
VPP	=	Voges Proskauer’s Performance
MRA	=	Methyl Red Activity
GA	=	Glucose Activity
MA	=	Mannitol Activity
LA	=	Lactose Activity
MA	=	Maltose Activity
PLT	=	Pyoluteorin
PRN	=	Pyrrolnitrin
PKSI	=	Polyketide Synthase
NRPS	=	Non-ribosomal Peptide Synthetases
HCN	=	Hydrogen Cyanide
Sfp	=	Surfactin Biosynthesis
SrfC	=	Surfactin Synthase
ItuD	=	Iturin A Biosynthesis
FenD	=	Fengycin Biosynthesis
BamC	=	Bacillomycin D
2,4-DAPG	=	2, 4-diacetyphloroglucinol
