# Supplementary material for: Isolation and Characterization of Root-Associated Bacterial Endophytes and Their Biocontrol Potential against Major Fungal Phytopathogens of Rice (Oryza sativa L.)
Source: Pathogens. 2020 Feb 28;9(3):172. doi: 10.3390/pathogens9030172 (PMC7157602; doi:10.3390/pathogens9030172)
Supplement: Supplementary file 1 [file pathogens-09-00172-s001.pdf]

pathogens

MDPI

Supplementary Material

Isolation and characterization of root-associated bacterial endophytes and their biocontrol potential against major fungal phytopathogens of rice (*Oryza sativa* L.)

Maqsood Ahmed Khaskheli [1], Lijuan Wu [1], Guoqing Chen [1], Long Chen [1], Sajid Hussain [1], Dawei Song [1], Sihui Liu [1] and Guozhong Feng [1,*]

[1] State Key Laboratory of Rice Biology, China National Rice Research Institute, Hangzhou, 311400, P. R. China; khaskheli.maqsood89@gmail.com (M.A.K); wulijuan@caas.cn (L.W.); chenguoqing@caas.cn (G.C.); chenlong@caas.cn (L.C.); hussainsajid@caas.cn (S.H.); 13051916600@163.com (D.S.); sihuil@126.com (S.L.);

* Correspondence: fengguozhong@caas.cn; (G.F.) Tel.: +86-57163370220; Fax: +86-57163370220

Table S1. List of used primers in the present study for detection of biosynthesis antibiotic related genes, from potential root-associated bacterial endophytes, by PCR amplifications.

S.No	Selected Antibiotics	Targeted genes	Primers	Sequences (5' to 3')	PCR amplifications	Expected Fragment Size (bp)
1	2, 4-diacetyphloroglu ci-nol	*2, 4-DAPG*	*Phl2f*<br>*Phl2r*	GAGGACGTCGAAGACCACCA<br>ACCGCAGCATCGTGTATGAG	Initial denaturation: 94 °C for 90 sec 94 °C for 35 sec, Renaturation: 53 °C for 30 sec, 72 °C for 45 sec, Final extension: 72 °C for 10 min	745
2	Pyoluteorin	*PLT*	*PltBf*<br>*PltBr*	CGGAGCATGGACCCCCAGC<br>GTGCCCGATATTGGTCTTGACCG AG	Initial denaturation: 94 °C for 2 min, 94 °C for 1 min, Renaturation: 58 °C for 45 sec, 72 °C for 1 min, Final extension: 72 °C for 10 min	773
**3**	Pyrrolnitrin	*PRN*	*Prncf*<br>*Prncr*	CCACAAGCCCGGCCAGGAGC<br>GAGAAGAGCGGGTCATGAAGCC	Initial denaturation: 94 °C for 2 min, 94 °C for 1 min, Renaturation: 58 °C for 45 sec, 72 °C for 1 min, Final extension : 72 °C for 10 min	719
**4**	Polyketide Synthase	*PKSI*	*Ksf*<br>*Ksr*	GCGATGGATCCCCAGCAGCG<br>GTGCCGGTCCCGTGCGTTTC	Initial denaturation: 95 °C for 5 min, Renaturation: 94 °C for 1 min, Annealing: 55 °C for 1 min, Extension: 72 °C for 1 min, Final extension: 72 °C for 10 min.	700
**5**	Non-ribosomal Peptide Synthetises	*NRPS*	*Nrpsf*<br>*Nrpsr*	GCCGGTGGTGCCTATGTCCC<br>CCCCGAATTTTCACTTG	Initial denaturation: 95 °C for 5 min, Renaturation: 94 °C for 1 min, Annealing: 55 °C for 1 min, Extension: 72 °C for 1 min, Final extension: 72 °C for 10 min.	1000
**6**	Hydrogen cyanide	*HCN*	*Pm2f*<br>*Pm7-26r*	TGCGGCATGGGCGTGTGCCATTG CTGCCTGG<br>CCGCTCTTGATCTGCAATTGCAG GCC	Initial denaturation: 94 °C for 2 min, 94 °C for 30 sec, Renaturation: 57 °C for 30 sec, 72 °C for 60 sec, Final extension: 72 °C for 10 min	570
**7**	Surfactin Biosynthesis	*Sfp*	*Sfp-f*<br>*Sfp-r*	ATGAAGATTTACGGAATTTA<br>TTATAAAAGCTCTTCGTACG	Initial denaturation: 95 °C for 5 min, Renaturation: 94 °C for 1 min, Annealing: 55 °C for 1 min, Extension: 72 °C for 1 min, Final extension: 72 °C for 10 min.	675
**8**	Iturin A Biosynthesis	*ItuD*	*ItuD1f*<br>*ItuD1r*	GATGCGATCTCCTTGGATGT<br>ATCGTCATGTGCTGCTTGAG	Initial denaturation: 95 °C for 5 min, Renaturation: 94 °C for 1 min, Annealing: 55 °C for 1 min, Extension: 72 °C for 1 min, Final extension: 72 °C for 10 min.	647
**9**	Fengycin Biosynthesis	*FenD*	*FenD1f*<br>*FenD1r*	TTTGGCAGCAGGAGAAGTTT<br>GCTGTCCGTTCTGCTTTTTC	Initial denaturation: 94 °C for 3 min, 94 °C for 1 min, Renaturation: 62 °C for 1 min, 72 °C for 1 min 45 sec, Final extension: 72 °C for 10 min	964
**10**	Surfactin Synthase	*SrfC*	*Sur3f*<br>*Sur3r*	ACAGTATGGAGGCATGGTC<br>TTCCGCCACTTTTTCAGTTT	Initial denaturation: 95 °C for 5 min, Renaturation: 94 °C for 1 min, Annealing: 55 °C for 1 min, Extension: 72 °C for 1 min, Final extension: 72 °C for 10 min.	441
**11**	Bacillomycin D	*BamC*	*Bacc1f*<br>*Bacc1r*	GAAGGACACGGCAGAGAGTC<br>CGCTGATGACTGTTCATGCT	Initial denaturation : 94 °C for 3 min 94 °C for 1 min, Renaturation: 60 °C for 30 sec, 72 °C for 1 min 45 sec, Final extension: 72 °C for 10 min	875
**12**	Cellulase	*Cellulase*	*CelBf*<br>*CelBr*	CCATGGATCATGAGGATGTGAA AACTC<br>CTCGAGTGAATTGGTTGTCTGAG CTG	Initial denaturation: 94 °C for 3 min, 94 °C for 30 sec, Renaturation: 51 °C for 30 sec, 72 °C for 1 min, Final extension: 72 °C for 10 min	1650

Note: (1). 2, 4-diacetyphloroglucinol (*2, 4-DAPG*), **(2).** Pyoluteorin (*PLT*), **(3).** Pyrrolnitrin (*PRN*), **(4).** Polyketide Synthase (*PKSI*), **(5).** Non-ribosomal Peptide synthetises (*NRPS*), **(6).** Hydrogen cyanide (*HCN*), **(7).** Surfactin Biosynthesis (*Sfp*), **(8).** Surfactin Synthase (*SrfC*), **(9).** Iturin A Biosynthesis (*ItuD*), **(10).** Fengycin Biosynthesis (*FenD*), **(11).** Bacillomycin D (*BamC*), **(12).** Cellulase.

Table S2. Identifications of biochemical and physiological characteristics of root-associated bacterial endophytes derived from rice host

Bacterial Endophytes	Biochemical and Physiological traits															
	GR	CA	EA	OA	GP	CA	MP	UA	NRA	VPP	MRA	SHA	GA	MA	LA	MA
B. marisflavi. C-1B-2	+	+	+	+	-	-	-	+	+	+	+	-	+	-	+	+
B. altitudinis. C-1B-3	+	+	+	+	+	-	-	+	-	+	+	+	+	+	+	-
L. fusiformis. C-1B-5	+	+	-	-	+	+	+	+	-	-	+	-	-	+	+	+
B. cereus. C-1B-6	+	+	+	+	+	+	+	+	+	+	+	+	+	+	+	+
B. cereus. C-1B-7	+	+	+	+	+	+	+	+	+	+	+	+	+	+	+	+
B. aryabhattai. C-1B-9	+	+	+	+	+	+	+	+	+	+	+	+	+	+	+	+
B. wiedmannii. C-1CL-2	+	-	+	-	-	+	+	+	-	+	+	+	-	+	+	+
B. wiedmannii. C-1CL-4	+	-	+	-	-	+	+	+	-	+	+	+	-	+	+	+
B. pseudomycoides. C-1F-2	+	+	-	+	+	-	-	+	-	+	+	+	-	+	+	-
B. wiedmannii. C-1F-3	+	-	+	-	-	+	+	+	-	+	+	+	-	+	+	+
B. thioparans. C-1G-1	+	+	-	+	-	-	+	+	+	+	+	-	+	+	+	+
B. altitudinis. C-1G-3	+	+	+	+	+	-	-	+	-	+	+	+	+	+	+	-
B. subterraneous. C-1S-1	+	+	+	+	+	+	+	+	+	+	+	+	+	+	+	+
B. lehensis. C-1S-2	+	+	+	+	+	+	+	+	+	+	+	+	+	+	+	+
F. phosphorivorans. C-1S-3	+	-	+	+	+	-	-	-	+	+	+	-	+	+	-	-
B. subterraneous. C-1S-4	+	+	+	+	+	+	+	+	+	+	+	+	+	+	+	+
B. acidiceler. C-1S-5	+	-	-	-	+	+	-	-	+	-	+	+	-	-	+	+
B. subterraneous. C-1ST-4	+	+	+	+	+	+	+	+	+	+	+	+	+	+	+	+
B. thioparans. C-1ST-5	+	+	+	+	+	+	+	+	+	+	+	+	+	+	+	+
L. fusiformis. C-1W-3	+	+	-	-	+	+	+	+	-	-	+	-	-	+	+	+
B. altitudinis. C-1W-5	+	+	+	+	+	-	-	+	-	+	+	+	+	+	+	-
B. altitudinis. C-1W-6	+	+	+	+	+	-	-	+	-	+	+	+	+	+	+	-
B. altitudinis. C-1W-14	+	+	+	+	+	-	-	+	-	+	+	+	+	+	+	-
B. aryabhattai. C-1WF-1	+	+	+	+	+	+	+	+	+	+	+	+	+	+	+	+
B. altitudinis. C-1WF-2	+	+	+	+	+	-	-	+	-	+	+	+	+	+	+	-
L. fusiformis. C-1WF-3	+	+	-	-	+	+	+	+	-	-	+	-	-	+	+	+
B. aryabhattai. C-1WF-7	+	+	+	+	+	+	+	+	+	+	+	+	+	+	+	+
B. aryabhattai. C-1WF-9	+	+	+	+	+	+	+	+	+	+	+	+	+	+	+	+
B. aryabhattai. C-1WNF-1	+	+	+	+	+	+	+	+	+	+	+	+	+	+	+	+
B. aryabhattai. C-1WNF-2	+	+	+	+	+	+	+	+	+	+	+	+	+	+	+	+
B. altitudinis. C-1WNF-7	+	+	+	+	+	-	-	+	-	+	+	+	+	+	+	-
B. aryabhattai. C-1WNF-8	+	+	+	+	+	+	+	+	+	+	+	+	+	+	+	+
B. aryabhattai. C-1WNF-9	+	+	+	+	+	+	+	+	+	+	+	+	+	+	+	+
F. phosphorivorans. C-1Y-1	+	-	+	+	+	-	-	-	+	+	+	-	+	+	-	-
F. nanhaiensis. C-1Y-2	+	+	-	+	-	+	+	+	+	-	+	+	+	+	+	+
B. marisflavi. C-1Y-4	+	+	+	+	+	-	-	-	+	+	+	+	-	+	-	+
F. nanhaiensis. C-1Y-7	+	+	-	+	-	+	+	+	+	-	+	+	+	+	+	+
F. phosphorivorans. C-1Y-8	+	-	+	+	+	-	-	-	+	+	+	-	+	+	-	-
F. phosphorivorans. C-1Y-9	+	-	+	+	+	-	-	-	+	+	+	-	+	+	-	-
F. phosphorivorans. C-1Y-10	+	-	+	+	+	-	-	-	+	+	+	-	+	+	-	-
F. nanhaiensis. C-1Y-11	+	+	-	+	-	+	+	+	+	-	+	+	+	+	+	+
F. nanhaiensis. C-1Y-12	+	+	-	+	-	+	+	+	+	-	+	+	+	+	+	+
F. phosphorivorans. C-1Y-13	+	-	+	+	+	-	-	-	+	+	+	-	+	+	-	-
F. phosphorivorans. C-1Y-16	+	-	+	+	+	-	-	-	+	+	+	-	+	+	-	-
F. phosphorivorans. C-1Y-17	+	-	+	+	+	-	-	-	+	+	+	-	+	+	-	-
P. alvei. C-2B-1	+	+	+	+	+	+	+	+	+	+	+	+	+	+	+	+
B. altitudinis. C-2B-2	+	+	+	+	+	-	-	+	-	+	+	+	+	+	+	-
B. aryabhattai. C-2 CL-1	+	+	+	+	+	+	+	+	+	+	+	+	+	+	+	+
B. altitudinis. C-2D-1	+	+	+	+	+	-	-	+	-	+	+	+	+	+	+	-
L. fusiformis. C-2GL-1	+	+	-	-	+	+	+	+	-	-	+	-	-	+	+	+
L. fusiformis. C-2GL-3	+	+	-	-	+	+	+	+	-	-	+	-	-	+	+	+
B. aryabhattai. C-2HW-1	+	+	+	+	+	+	+	+	+	+	+	+	+	+	+	+
B. cereus. C-2HW-2	+	+	+	+	+	+	+	+	+	+	+	+	+	+	+	+
L. mangiferihumi. C-2HW-3	+	+	+	+	+	+	+	+	+	+	+	+	+	+	+	+
B. aryabhattai. C-2HW-4	+	+	+	+	+	+	+	+	+	+	+	+	+	+	+	+
B. aryabhattai. C-2HW-5	+	+	+	+	+	+	+	+	+	+	+	+	+	+	+	+
B. altitudinis. C-2HW-6	+	+	+	+	+	-	-	+	-	+	+	+	+	+	+	-
P. alvei. C-2HW-7	+	+	+	+	+	+	+	+	+	+	+	+	+	+	+	+
B. aryabhattai. C-2LY-1	+	+	+	+	+	+	+	+	+	+	+	+	+	+	+	+
B. marisflavi. C-2LY-2	+	+	+	+	-	-	-	+	+	+	+	-	+	-	+	+
L. fusiformis. C-2LY-4	+	+	-	-	+	+	+	+	-	-	+	-	-	+	+	+

B. marisflavi. C-2LY-5	+	+	+	+	-	-	-	+	+	+	+	-	+	-	+	+
B. aryabhattai. C-2LY-6	+	+	+	+	+	+	+	+	+	+	+	+	+	+	+	+
B. aryabhattai. C-2LY-7	+	+	+	+	+	+	+	+	+	+	+	+	+	+	+	+
B. altitudinis. C-2R-1	+	+	+	+	+	-	-	+	-	+	+	+	+	+	+	-
B. altitudinis. C-2RO-1	+	+	+	+	+	-	-	+	-	+	+	+	+	+	+	-
B. altitudinis. C-2RO-3	+	+	+	+	+	-	-	+	-	+	+	+	+	+	+	-
B. altitudinis. C-2RO-4	+	+	+	+	+	-	-	+	-	+	+	+	+	+	+	-
B. altitudinis. C-2S-1	+	+	+	+	+	-	-	+	-	+	+	+	+	+	+	-
B. cereus. C-2SN-1	+	+	+	+	+	+	+	+	+	+	+	+	+	+	+	+
B. altitudinis. C-2SN-2	+	+	+	+	+	-	-	+	-	+	+	+	+	+	+	-
B. altitudinis. C-2SN-3	+	+	+	+	+	-	-	+	-	+	+	+	+	+	+	-
B. altitudinis. C-2SP-1	+	+	+	+	+	-	-	+	-	+	+	+	+	+	+	-
P. cucumis. C-2T-1	+	+	+	+	+	-	-	+	+	+	+	+	-	+	-	+
B. cereus. C-2W-1	+	+	+	+	+	+	+	+	+	+	+	+	+	+	+	+
B. cereus. C-2W-2	+	+	+	+	+	+	+	+	+	+	+	+	+	+	+	+
B. tequilensis. C-2W-3	+	+	+	-	-	+	+	-	-	+	+	-	+	+	+	+
B. cereus. C-2W-4	+	+	+	+	+	+	+	+	+	+	+	+	+	+	+	+
B. cereus. C-2W-5	+	+	+	+	+	+	+	+	+	+	+	+	+	+	+	+
B. indicus. C-2Y-1	+	+	+	+	+	+	+	+	+	+	+	+	+	+	+	+
B. marisflavi. C-2Y-2	+	+	+	+	-	-	-	+	+	+	+	-	+	-	+	+
B. altitudinis. C-2Y-3	+	+	+	+	+	-	-	+	-	+	+	+	+	+	+	-
B. altitudinis. C-2Y-4	+	+	+	+	+	-	-	+	-	+	+	+	+	+	+	-
B. cereus. C-2Y-5	+	+	+	+	+	+	+	+	+	+	+	+	+	+	+	+
B. marisflavi. C-2Y-6	+	+	+	+	-	-	-	+	+	+	+	-	+	-	+	+
B. wiedmannii. C-3CL-1	+	-	+	-	-	+	+	+	-	+	+	+	-	+	+	+
B. wiedmannii. C-3CL-3	+	-	+	-	-	+	+	+	-	+	+	+	-	+	+	+
B. wiedmannii. C-3CL-4	+	-	+	-	-	+	+	+	-	+	+	+	-	+	+	+
B. cereus. C-3CL-5	+	+	+	+	+	+	+	+	+	+	+	+	+	+	+	+
B. cereus. C-3CL-6	+	+	+	+	+	+	+	+	+	+	+	+	+	+	+	+
B. toyonensis. C-3CL-7	+	+	-	+	+	-	-	+	-	+	+	+	+	-	+	+
B. wiedmannii. C-3CL-8	+	-	+	-	-	+	+	+	-	+	+	+	-	+	+	+
L. mangiferihumi. C-3F-5	+	+	+	+	+	+	+	+	+	+	+	+	+	+	+	+
B. altitudinis. C-3F-7	+	+	+	+	+	-	-	+	-	+	+	+	+	+	+	-
L. fusiformis. C-3F-8	+	+	-	-	+	+	+	+	-	-	+	-	-	+	+	+
B. wiedmannii. C-3G-1	+	-	+	-	-	+	+	+	-	+	+	+	-	+	+	+
C. metallidurans. C-3P-3	-	+	-	+	-	+	+	+	-	-	-	+	+	+	+	+
C. metallidurans. C-3P-5	-	+	-	+	-	+	+	+	-	-	-	+	+	+	+	+
B. altitudinis. C-3R-1	+	+	+	+	+	-	-	+	-	+	+	+	+	+	+	-
B. aryabhattai. C-3R-2	+	+	+	+	+	+	+	+	+	+	+	+	+	+	+	+
B. altitudinis. C-3R-3	+	+	+	+	+	-	-	+	-	+	+	+	+	+	+	-
B. altitudinis. C-3R-5	+	+	+	+	+	-	-	+	-	+	+	+	+	+	+	-
B. altitudinis. C-3R-6	+	+	+	+	+	-	-	+	-	+	+	+	+	+	+	-
L. fusiformis. C-3R-7	+	+	-	-	+	+	+	+	-	-	+	-	-	+	+	+
B. altitudinis. C-3R-8	+	+	+	+	+	-	-	+	-	+	+	+	+	+	+	-
B. cereus. C-3R-9	+	+	+	+	+	+	+	+	+	+	+	+	+	+	+	+
B. altitudinis. C-3R-10	+	+	+	+	+	-	-	+	-	+	+	+	+	+	+	-
C. metallidurans. C-3SP-3	-	+	-	+	-	+	+	+	-	-	-	+	+	+	+	+
C. metallidurans. C-3T-1	-	+	-	+	-	+	+	+	-	-	-	+	+	+	+	+
C. metallidurans. C-3T-2	-	+	-	+	-	+	+	+	-	-	-	+	+	+	+	+
C. metallidurans. C-3T-3	-	+	-	+	-	+	+	+	-	-	-	+	+	+	+	+
P. uliginis. C-3T-4	+	+	+	+	+	+	+	+	+	+	+	+	+	+	+	+
C. metallidurans. C-3T-5	-	+	-	+	-	+	+	+	-	-	-	+	+	+	+	+
C. metallidurans. C-3T-6	-	+	-	+	-	+	+	+	-	-	-	+	+	+	+	+
P. cucumis. C-3T-7	+	+	+	+	+	-	-	+	+	+	+	+	-	+	-	+
C. metallidurans. C-3T-8	-	+	-	+	-	+	+	+	-	-	-	+	+	+	+	+
M. laevaniformans. C-3T-9	-	+	+	-	+	+	-	+	-	-	+	-	+	+	+	+
L. fusiformis. C-3W-3	+	+	-	-	+	+	+	+	-	-	+	-	-	+	+	+
B. altitudinis. C-3WA-8	+	+	+	+	+	-	-	+	-	+	+	+	+	+	+	-
B. marisflavi. C-3Y-2	+	+	+	+	-	-	-	+	+	+	+	-	+	-	+	+
B. marisflavi. C-3Y-5	+	+	+	+	-	-	-	+	+	+	+	-	+	-	+	+
F. phosphorivorans. C-3Y-10	+	-	+	+	+	-	-	-	+	+	+	-	+	+	-	-

Note: Gram reaction **(GR)**, Catalase activity **(CA)**, Endospore activity **(EA)**, Oxidase activity **(OA)**, Gelatinase production**(GP)**, Citrate activity **(CA)**, Motility performance **(MP)**, Urease activity **(UA)**, Nitrate reduction activity **(NRA)**, Voges proskauer's production **(VPP)**, Methyl red activity **(MRA)**, Starch hydrolyses activity **(SHA)**, Glucose activity **(GA)**, Maltose activity **(MA)**, Lactose activity **(LA)**, Mannitol activity **(MA)**. **(+)** = Indicate positive presences, **(-)** = Indicate negative presences.

Table S3. Molecularly determination of functional biosynthesis antibiotic related genes from root-associated bacterial endophytes

**Bacterial Endophytes**	**Antibiotic Producing Genes**											
	**2,4-DAPG**	**PLT**	**PRN**	**PKSI**	**NRPS**	**HCN**	**Sfp**	**SrfC**	**ItuD**	**FenD**	**BamC**	**Cellulase**
B. marisflavi C-1B-2	+	+	+	+	+	+	+	+	+	+	+	+
B. altitudinis C-1B-3	+	+	+	+	+	+	+	+	+	+	+	+
L. fusiformis C-1B-5	-	+	-	-	+	-	-	+	-	+	+	-
B. cereus C-1B-6	+	+	+	+	+	+	+	+	+	+	+	+
B. cereus C-1B-7	+	+	+	+	+	+	+	+	+	+	+	+
B. aryabhattai C-1B-9	+	+	+	+	+	+	+	+	+	+	+	+
B. wiedmannii C-1CL-2	+	+	+	+	+	+	+	+	+	+	+	+
B. wiedmannii C-1CL-4	+	+	+	+	+	+	+	+	+	+	+	+
B. pseudomycoides C-1F-2	-	+	+	-	+	-	+	+	-	+	+	-
B. wiedmannii C-1F-3	+	+	+	+	+	+	+	+	+	+	+	+
B. thioparans C-1G-1	-	+	+	+	-	+	-	+	-	-	-	+
B. altitudinis C-1G-3	+	+	+	+	+	+	+	+	+	+	+	+
B. subterraneous C-1S-1	+	-	+	+	+	-	-	-	-	+	+	+
B. lehensis C-1S-2	+	+	+	-	-	+	+	+	-	+	+	-
F. phosphorivorans C-1S-3	-	+	-	-	+	+	-	+	+	-	-	+
B. subterraneous C-1S-4	+	-	+	+	+	-	-	-	-	+	+	+
B. acidiceler C-1S-5	-	-	+	+	+	-	-	-	+	+	+	-
B. subterraneous C-1ST-4	+	-	+	+	+	-	-	-	-	+	+	+
B. thioparans C-1ST-5	-	+	+	+	-	+	-	+	-	-	-	+
L. fusiformis C-1W-3	-	+	-	-	+	-	-	+	-	+	+	-
B. altitudinis C-1W-5	+	+	+	+	+	+	+	+	+	+	+	+
B. altitudinis C-1W-6	+	+	+	+	+	+	+	+	+	+	+	+
B. altitudinis C-1W-14	+	+	+	+	+	+	+	+	+	+	+	+
B. aryabhattai C-1WF-1	+	+	+	+	+	+	+	+	+	+	+	+
B. altitudinis C-1WF-2	+	+	+	+	+	+	+	+	+	+	+	+
L. fusiformis C-1WF-3	-	+	-	-	+	-	-	+	-	+	+	-
B. aryabhattai C-1WF-7	+	+	+	+	+	+	+	+	+	+	+	+
B. aryabhattai C-1WF-9	+	+	+	+	+	+	+	+	+	+	+	+
B. aryabhattai C-1WNF-1	+	-	+	+	+	+	+	+	+	+	+	+
B. aryabhattai C-1WNF-2	+	+	+	+	+	+	+	+	+	+	+	+
B. altitudinis C-1WNF-7	+	+	+	+	+	+	+	+	+	+	+	+
B. aryabhattai C-1WNF-8	+	+	+	+	+	+	+	+	+	+	+	+
B. aryabhattai C-1WNF-9	+	+	+	+	+	+	+	+	+	+	+	+
F. phosphorivorans C-1Y-1	-	+	-	-	+	+	-	+	+	-	-	+
F. nanhaiensis C-1Y-2	-	-	+	+	-	-	+	+	+	-	+	-
B. marisflavi C-1Y-4	+	+	+	+	+	+	+	+	+	+	+	+
F. nanhaiensis C-1Y-7	-	-	+	+	-	-	+	+	+	-	+	-
F. phosphorivorans C-1Y-8	-	+	-	-	+	+	-	+	+	-	-	+
F. phosphorivorans C-1Y-9	-	+	-	-	+	+	-	+	+	-	-	+
F. phosphorivorans C-1Y-10	-	+	-	-	+	+	-	+	+	-	-	+
F. nanhaiensis C-1Y-11	-	-	+	+	-	-	+	+	+	-	+	-
F. nanhaiensis C-1Y-12	-	-	+	+	-	-	+	+	+	-	+	-
F. phosphorivorans C-1Y-13	-	+	-	-	+	+	-	+	+	-	-	+
F. phosphorivorans C-1Y-16	-	+	-	-	+	+	-	+	+	-	-	+
F. phosphorivorans C-1Y-17	-	+	-	-	+	+	-	+	+	-	-	+
P. alvei C-2B-1	+	+	-	-	+	-	+	-	+	-	+	-
B. altitudinis C-2B-2	+	+	+	+	+	+	+	+	+	+	+	+
B. aryabhattai C-2CL-1	+	+	+	+	+	+	+	+	+	+	+	+
B. altitudinis C-2D-1	+	+	-	+	+	+	+	+	+	+	+	+
L. fusiformis C-2GL-1	-	+	-	-	+	-	-	+	-	+	+	-
L. fusiformis C-2GL-3	-	+	-	-	+	-	-	+	-	+	+	-
B. aryabhattai C-2HW-1	+	+	+	+	+	+	+	+	+	+	+	+
B. cereus C-2HW-2	+	+	+	+	+	+	+	+	+	+	+	+
L. mangiferihumi C-2HW-3	-	-	+	+	-	+	+	+	-	+	+	-
B. aryabhattai C-2HW-4	+	+	+	+	+	+	+	+	+	+	+	+
B. aryabhattai C-2HW-5	+	+	+	+	+	+	+	+	+	+	+	+
B. altitudinis C-2HW-6	+	+	-	+	+	+	+	+	+	+	+	+
P. alvei C-2HW-7	+	+	-	-	+	-	+	-	+	-	+	-
B. aryabhattai C-2LY-1	+	+	+	+	+	+	+	+	+	+	+	+
B. marisflavi C-2LY-2	+	+	+	+	+	+	+	+	+	+	+	+
L. fusiformis C-2LY-4	-	+	-	-	+	-	-	+	-	+	+	-
B. marisflavi C-2LY-5	+	+	+	+	+	+	+	+	+	+	+	+

B. aryabhattai C-2LY-6	+	+	+	+	+	+	+	+	+	+	+	+
B. aryabhattai C-2LY-7	+	+	+	+	+	+	+	+	+	+	+	+
B. altitudinis C-2R-1	+	+	+	+	+	+	+	+	+	+	+	+
B. altitudinis C-2RO-1	+	+	+	+	+	+	+	+	+	+	+	+
B. altitudinis C-2RO-3	+	+	+	+	+	+	+	+	+	+	+	+
B. altitudinis C-2RO-4	+	+	+	+	+	+	+	+	+	+	+	+
B. altitudinis C-2S-1	+	+	+	+	+	+	+	+	+	+	+	+
B. cereus C-2SN-1	+	+	+	+	+	+	+	+	+	+	+	+
B. altitudinis C-2SN-2	+	+	+	+	+	+	+	+	+	+	+	+
B. altitudinis C-2SN-3	+	+	+	+	+	+	+	+	+	+	+	+
B. altitudinis C-2SP-1	+	+	+	+	+	+	+	+	+	+	+	+
P. cucumis C-2T-1	-	+	+	+	-	-	+	-	+	+	+	+
B. cereus C-2W-1	+	+	+	+	+	+	+	+	+	+	+	+
B. cereus C-2W-2	+	+	+	+	+	+	+	+	+	+	+	+
B. tequilensis C-2W-3	+	+	-	-	+	+	-	-	+	+	+	+
B. cereus C-2W-4	+	+	+	+	+	+	+	+	+	+	+	+
B. cereus C-2W-5	+	+	+	+	+	+	+	+	+	+	+	+
B. indicus C-2Y-1	+	+	-	+	+	+	+	+	+	+	+	+
B. marisflavi C-2Y-2	+	+	+	+	+	+	+	+	+	+	+	+
B. altitudinis C-2Y-3	+	+	+	+	+	+	+	+	+	+	+	+
B. altitudinis C-2Y-4	+	+	+	+	+	+	+	+	+	+	+	+
B. cereus C-2Y-5	+	+	+	+	+	+	+	+	+	+	+	+
B. marisflavi C-2Y-6	+	+	+	+	+	+	+	+	+	+	+	+
B. wiedmannii C-3CL-1	+	+	+	+	+	+	+	+	+	+	+	+
B. wiedmannii C-3CL-3	+	+	+	+	+	+	+	+	+	+	+	+
B. wiedmannii C-3CL-4	+	+	+	+	+	+	+	+	+	+	+	+
B. cereus C-3CL-5	+	+	+	+	+	+	+	+	+	+	+	+
B. cereus C-3CL-6	+	+	+	+	+	+	+	+	+	+	+	+
B. toyonensis C-3CL-7	+	-	-	+	+	-	+	-	+	-	+	+
B. wiedmannii C-3CL-8	+	+	+	+	+	+	+	+	+	+	+	+
L. mangiferihumi C-3F-5	-	-	+	+	-	+	+	+	-	+	+	-
B. altitudinis C-3F-7	+	+	-	+	+	+	+	+	+	+	+	+
L. fusiformis C-3F-8	-	+	-	-	+	-	-	+	-	+	+	-
B. wiedmannii C-3G-1	+	+	-	+	+	+	+	+	+	+	+	+
C. metallidurans C-3P-3	+	+	-	-	-	+	+	+	+	+	+	-
C. metallidurans C-3P-5	+	+	-	-	-	+	+	+	+	+	+	-
B. altitudinis C-3R-1	+	+	+	+	+	+	+	+	+	+	+	+
B. aryabhattai C-3R-2	+	+	+	+	+	+	+	+	+	+	+	+
B. altitudinis C-3R-3	+	+	+	+	+	+	+	+	+	+	+	+
B. altitudinis C-3R-5	+	+	+	+	+	+	+	+	+	+	+	+
B. altitudinis C-3R-6	+	+	+	+	+	+	+	+	+	+	+	+
L. fusiformis C-3R-7	-	+	-	-	+	-	-	+	-	+	+	-
B. altitudinis C-3R-8	+	+	+	+	+	+	+	+	+	+	+	+
B. cereus C-3R-9	+	+	+	+	+	+	+	+	+	+	+	+
B. altitudinis C-3R-10	+	+	+	+	+	+	+	+	+	+	+	+
C. metallidurans C-3SP-3	+	+	-	-	-	+	+	+	+	+	+	-
C. metallidurans C-3T-1	+	+	-	-	-	+	+	+	+	+	+	-
C. metallidurans C-3T-2	+	+	-	-	-	+	+	+	+	+	+	-
C. metallidurans C-3T-3	+	+	-	-	-	+	+	+	+	+	+	-
P. uliginis C-3T-4	+	+	+	+	+	+	+	+	+	+	+	+
C. metallidurans C-3T-5	+	+	-	-	-	+	+	+	+	+	+	-
C. metallidurans C-3T-6	+	+	-	-	-	+	+	+	+	+	+	-
P. cucumis C-3T-7	-	+	+	+	-	-	+	-	+	+	+	+
C. metallidurans C-3T-8	+	+	-	-	-	+	+	+	+	+	+	-
M. laevaniformans C-3T-9	-	+	-	+	+	-	+	-	+	+	+	+
L. fusiformis C-3W-3	-	+	-	-	+	-	-	+	-	+	+	-
B. altitudinis C-3WA-8	+	+	+	+	+	+	+	+	+	+	+	+
B. marisflavi C-3Y-2	+	+	+	+	+	+	+	+	+	+	+	+
B. marisflavi C-3Y-5	+	+	+	+	+	+	+	+	+	+	+	+
F. phosphorivorans C-3Y-10	-	+	-	-	+	+	-	+	+	-	-	+

Note: (1). 2, 4-diacetyphloroglucinol (*2, 4-DAPG*), **(2)**. Pyoluteorin (*PLT*), **(3)** Pyrrolnitrin (*PRN*), **(4)** Polyketide Synthase (*PKSI*), **(5)** Non-ribosomal Peptide synthetises (*NRPS*), **(6)** Hydrogen cyanide (*HCN*), **(7)** Surfactin Biosynthesis (*Sfp*), **(8)** Surfactin Synthase (*SrfC*), **(9)** Iturin A Biosynthesis (*ItuD*), **(10)** Fengycin Biosynthesis (*FenD*), **(11)** Bacillomycin D (*BamC*), **(12)** Cellulase. **(+)** = Indicate positive presences, **(-) =** Indicate negative presences.
